# GOULD EDU: Cluster-Randomized Trial of an Educational Intervention to Improve Lipid-Lowering Guideline Adherence

**DOI:** 10.1016/j.jacadv.2025.102403

**Published:** 2025-12-03

**Authors:** Christopher P. Cannon, Christie M. Ballantyne, Deepak L. Bhatt, James A. de Lemos, Qi Gao, Naishu Kui, Robert S. Rosenson, Katherine E. Mues, Jason Exter, Shushama Alam, Mikhail N. Kosiborod, GOULD EDU Investigators

**Affiliations:** a Cardiovascular Division, Brigham and Women's Hospital, Harvard Medical School, Boston, Massachusetts, USA; b Baylor College of Medicine and the Texas Heart Institute, Houston, Texas, USA; c Mount Sinai Fuster Heart Hospital, Icahn School of Medicine at Mount Sinai, New York, New York, USA; d University of Texas Southwestern Medical Center, Dallas, Texas, USA; e Baim Institute for Clinical Research, Boston, Massachusetts, USA; f Icahn School of Medicine at Mount Sinai, New York, New York, USA; g Amgen Inc., Thousand Oaks, California, USA; h Saint Luke's Mid America Heart Institute, University of Missouri-Kansas City, Kansas City, Missouri, USA

**Keywords:** atherosclerotic cardiovascular disease, LDL-C goals, lipid-lowering therapy, myocardial infarction, observational registry, proprotein convertase subtilisin/kexin type 9 inhibitor

## Abstract

**Background:**

Adherence to lipid-lowering therapy (LLT) guidelines is suboptimal, leading to poor achievement of low-density lipoprotein cholesterol (LDL-C) goals and elevated risk for atherosclerotic cardiovascular disease (ASCVD) events.

**Objectives:**

The purpose of this study was to determine whether an educational intervention targeting physicians improves optimization of LLT over 1 year for patients with ASCVD.

**Methods:**

GOULD (Getting to an Improved Understanding of Low-Density Lipoprotein Cholesterol and Dyslipidemia Management) EDU (Education) was a 1-year extension in a subset of sites in the GOULD prospective, observational registry of United States patients with ASCVD. In a cluster-randomized, stepped-wedge design, sites were randomized to 2 waves of an educational intervention that included 2 webinars and access to interactive data reports showing site-specific performance alongside full-study data over the course of the study. Chart reviews assessed LLTs and patient outcomes. Outcomes assessed over 1 year included change in prescribed LLT, frequency of measurement of LDL-C, LDL-C levels, LDL-C goal achievement, and responses on a structured physician questionnaire.

**Results:**

Thirty sites were randomized to Step 1 (n = 616) and 26 sites to Step 2 (n = 382). The mean (SD) age was 70.2 (9.6) years, 61.1% (610/998) were male, and 85.3% (851/998) were White. During the study, 83.6% (820/981) of patients had no change in LLT, and intensification occurred in only 7.0% (69/981); intensification did not differ by intervention step. The percentage of patients who met the goal of LDL-C <70 mg/dL was 39.0% (202/518) at baseline and 39.5% (252/638) at 1 year.

**Conclusions:**

An educational intervention with physicians had little impact on LLT optimization or LDL-C goal achievement in patients with ASCVD over a 1-year period. Novel approaches are needed to improve LDL-C goal achievement. (Getting to an Improved Understanding of Low-Density Lipoprotein Cholesterol and Dyslipidemia Management [GOULD] a Registry of High Cardiovascular Risk Subjects in the United States; NCT02993120)

Despite extensive evidence accumulated over decades that low-density lipoprotein cholesterol (LDL-C)-lowering is an effective means of reducing atherosclerotic cardiovascular disease (ASCVD) risk,^1^^,^^2^ many studies have documented the underutilization of lipid-lowering therapy (LLT), even in the highest-risk patients.^3^, ^4^, ^5^, ^6^ The substantial and persistent gap between guidelines and real-world treatment patterns leaves patients at unnecessary ASCVD risk.

Statin treatment is the mainstay of LLT, but many patients do not achieve LDL-C goals with statins alone. The 2018 American Heart Association/American College of Cardiology Multisociety Guideline on the Management of Blood Cholesterol^1^ recommends that very high-risk patients who are receiving maximum-tolerated statin therapy and are unable to reach LDL-C <70 mg/dL should have intensification of LLT. The 2019 European Society of Cardiology/European Atherosclerosis Society guidelines^7^ and American College of Cardiology Expert Consensus Decision Pathway^8^ suggest a lower LDL-C goal, with intensification when LDL-C is ≥55 mg/dL.

The GOULD (Getting to an Improved Understanding of Low-Density Lipoprotein Cholesterol and Dyslipidemia Management) study followed patients with ASCVD receiving any LLT in order to assess treatment patterns, LDL-C goal achievement, and frequency of lipid testing.^4^ During 2 years of follow-up, only 17% of patients had intensification of LLT, with only one-third of patients achieving LDL-C <70 mg/dL.^4^ Over the same period, 11% of all patients never had a lipid panel conducted, and 21% of patients had only 1 lipid panel conducted.

Although the overall results regarding LLT guideline adherence were discouraging in GOULD, LLT intensification rates were somewhat greater for physicians who were at teaching hospitals, had lipid protocols in place at their institutions, were cardiologists, or stated that the ideal LDL-C goal for ASCVD patients was <70 mg/dL.^4^ Building on these factors that may lead to better guideline adherence, GOULD EDU (Education) was initiated to test the effectiveness of a tailored educational intervention to encourage physicians to improve guideline-consistent intensification of LLT, with the ultimate aim of optimizing their patients’ LDL-C levels and reducing their ASCVD risk.

## Methods

### Design and patients

GOULD EDU was a 1-year extension of GOULD,^4^ a prospective, observational registry of U.S. patients with ASCVD who were taking any LLT and followed for 2 years. In GOULD EDU, prospective data collection continued as in the parent study, and sites were approached to participate in an educational intervention, administered in a cluster-randomized, stepped-wedge design. Participating sites were randomized to receive the intervention in either Step 1 (at the start of the first 6-month period of the extension) or Step 2 (at the start of the second 6-month period of the extension) (Supplemental Figure 1). The stepped-wedge approach was used to account for secular trends that might impact LDL-C goals or LLT prescribing independently of the educational intervention.

Patients had been enrolled in the GOULD parent study^4^ with eligibility criteria of age ≥18 years and established ASCVD (history of myocardial infarction, coronary artery disease, coronary or other arterial revascularization, ischemic stroke or transient ischemic attack, carotid artery stenosis, or documented peripheral arterial disease secondary to atherosclerosis [aortic aneurysm, ankle-brachial index <0.9, imaging evidence of >50% stenosis in any peripheral artery, or intermittent claudication]). All patients were to have received stable LLT for at least 4 weeks prior to enrollment in GOULD.

Eligible patients were enrolled into 1 of 3 cohorts at the GOULD study baseline, and patients remained in the same cohorts for GOULD EDU per their GOULD baseline status: 1) patients currently receiving a proprotein convertase subtilisin/kexin type 9 inhibitor (PCSK9i); 2) patients who were not receiving PCSK9i treatment and had LDL-C ≥100 mg/dL; and 3) patients who were not receiving PCSK9i treatment and had LDL-C of 70 to 99 mg/dL.

Patients who completed 2 years of participation in GOULD and provided informed consent to participate in the extension study were eligible for GOULD EDU.

Each site obtained Institutional Review Board approval, and the study was performed in accordance with Good Clinical Practice guidelines.

### Educational intervention

For all participating sites, the principal investigator and/or study coordinator participated in the educational intervention, which consisted of 2 educational webinars designed and led by members of the GOULD steering committee and access to an Interactive GOULD Registry Site Report. Participants were encouraged to share the site reports with their staff. The webinars discussed recent LLT guidelines, patterns of LLT, and LDL-C control in the United States, an overview of results from the primary GOULD study, and opportunities for improvement in clinical care. For Step 1 sites, the webinars occurred in September and October of 2020, shortly after the GOULD EDU began. For Step 2 sites, they occurred in January and February of 2021.

The Interactive GOULD Registry Site Report contained data on LLT patterns and LDL-C measurements from GOULD EDU sites that were updated 4 times during the study. Sites in Step 1 and Step 2 received access to the site reports in conjunction with the timing of their respective webinars. Site personnel could view data from their own site benchmarked to data from the full GOULD EDU cohort. The report presented the data in various ways, and users could drill down for additional details, including enrollment across sites, LDL-C changes over time, LDL-C goal achievement (<70 mg/dL and <55 mg/dL) over time, percentages of patients receiving various LLTs, percentages of patients receiving guideline-recommended therapy for selected subgroups of patients, distribution of demographic and disease characteristics, and rates of cardiovascular (CV) events. Examples of data shown in the Interactive GOULD Registry Site Report are shown in Supplemental Figure 2. Sites were encouraged to share the report with other practitioners at their site at staff meetings.

### Study procedures

#### Chart reviews

Chart reviews were performed by research team personnel at the enrolling sites. Baseline demographic and disease characteristics for GOULD EDU were captured in the last chart review during the GOULD parent study, between July 1, 2019, and the start of GOULD EDU on June 30, 2020. Patients enrolled in GOULD EDU underwent another chart review at the end of GOULD EDU, which covered the first and second halves (referred to as Periods 1 and 2) of the 1-year study from July 2020 to July 2021. During all chart reviews, the most recent values were recorded in the electronic case report forms for changes in lipid-lowering medications, lipid laboratory values, CV-related events, medical history, and safety data. Dates of medication changes, laboratory tests, and relevant events were captured in order to track changes over time.

#### Physician questionnaire

At the beginning (September 2020) and end (July 2021) of GOULD EDU, the principal investigator at each center was asked by email to complete an online questionnaire regarding their awareness and application of American College of Cardiology/American Heart Association and European Society of Cardiology LLT guidelines, their perceptions of barriers to intensifying LLT, and their own patterns of use of LLTs. The questionnaire had 54 items that were mostly multiple-choice or yes/no answers. The same questionnaire was used for the pre- and post-educational intervention survey.

### Outcome variables

Key patient-level outcomes included the percentage of patients who attained lipid-lowering goals of LDL-C <70 mg/dL and LDL-C <55 mg/dL and the percentage of patients who had intensification of LDL-C-lowering therapy. In addition, patterns of usage of various LLTs at baseline and over time during GOULD EDU were evaluated, including the percentages of patients initiating or discontinuing statin, ezetimibe, or PCSK9i therapy; increasing or decreasing the dose or switching to a different type of statin or PCSK9i; changes in other LLTs (including fish oil/omega-3 preparations, bile acid sequestrants, mipomersen, lomitapide, apheresis, or any new LLT); or no changes in LLT. Use of intensive LLT was also assessed and was defined as use of high-intensity statin or use of any statin plus ezetimibe or use of PCSK9i. Percentage of patients who had lipids measured and frequency of lipid measurement were also assessed.

Physician-level outcomes were preintervention vs postintervention responses on the physician questionnaire, measured as the percentage of physicians selecting each response.

### Statistical methods

Descriptive statistics were used to summarize all outcomes. Continuous variables were summarized as mean, median, SD, and IQR. Categorical variables were summarized by number and percent. Outcomes were generally summarized by the patient enrollment cohorts from GOULD (receiving PCSK9, no PCSK9i and LDL-C ≥100 mg/dL, no PCSK9i and LDL-C 70-99 mg/dL) and the intervention step in which the site participated (Step 1 vs Step 2). Results were also summarized by Period 1 vs 2 (first half vs last half of the study). LLTs were summarized by study month to capture changes over time.

To assess the benefits of the educational intervention on LLT intensification, LDL-C levels, and goal achievement, outcomes were evaluated for patients in intervention Step 1 vs Step 2 and comparing across study periods (Period 1 vs Period 2).

All analyses were performed using SAS version 9.4 (SAS Institute Inc).

## Results

Of the original 119 U.S. study sites, 56 participated in GOULD EDU with 30 sites randomized to Step 1 and 26 sites to Step 2. Of 2,439 patients enrolled at these sites who completed the original 2-year GOULD registry, 998 patients consented to enroll in GOULD EDU (Step 1: n = 616; Step 2: n = 382) (Supplemental Figure 3), and chart review was completed in 97.6% (974/998) of patients, with follow-up for a median of 352 (IQR: 337-380) days. Attendance at the webinars in both steps was just 40 to 55% for lead study physicians, but 100% for the study coordinator of each site.

### Site and physician characteristics

Most patients were at sites that were nonhospital based (84.9%, 847/998), were not teaching centers (81.2%, 810/998), were in urban settings (79.6%, 794/998), and used electronic records (73%, 729/998). Lipid management protocols were in place for the sites at which 43.2% (431/998) of patients were treated. Demographics of the lead study physicians (weighted by number of patients at each site where a physician is counted for each subject they treat) showed a mean age of 58.6 (SD: 10.2) years, and 82.9% (792/955) were male. Patients were enrolled at sites where the lead physicians specialized in cardiology (61.9%, 618/998) and internal medicine (41%, 409/998), with a smaller number in endocrinology (4.8%, 48/998), nephrology (0.9%, 9/998), or another specialty (4.1%, 41/998).

### Patient baseline characteristics

At baseline of GOULD EDU, patients (N = 998) had a mean (SD) age of 70.2 (9.6) years, 61.1% (610/998) were male, 85.3% (851/998) were White, 83.7% (835/998) had coronary artery disease, and 35.2% (351/998) had type 2 diabetes mellitus (Table 1). Baseline characteristics are reported by the cohorts designated at the start of GOULD; median (quartile 1, quartile 3 [Q1, Q3]) LDL-C was 89.0 (64.0, 113.0) mg/dL in the LDL-C ≥100 cohort, 74.0 (60.0, 89.0) mg/dL in the LDL-C 70 to 99 cohort, and 62.0 (40.0, 90.0) mg/dL in the PCSK9i cohort. Characteristics of patients were generally similar for patients in Step 1 and Step 2 (Supplemental Table 1), and characteristics at baseline of the GOULD parent study were similar in patients who did or did not choose to participate in GOULD EDU (Supplemental Table 2).

**Table 1:** Demographic and Disease Characteristics From Baseline of GOULD EDU

	Cohorts as Designated at Baseline in the GOULD Parent Study^a^			All Patients (N = 998)
	Patients on PCSK9i mAb at Baseline (n = 125)	Patients Not on PCSK9i at Baseline		
		LDL-C ≥100 mg/dL at Baseline (n = 355)	LDL-C 70–99 mg/dL at Baseline (n = 518)	
Age (y), mean (SD)	70.3 (9.7)	68.8 (9.7)	71.1 (9.4)	70.2 (9.6)
Male	57.6% (72)	53.5% (190)	67.2% (348)	61.1% (610)
Ethnicity				
Hispanic or Latino	4.8% (6)	13.5% (48)	7.5% (39)	9.3% (93)
Not Hispanic or Latino	93.6% (117)	86.5% (307)	92.5% (479)	90.5% (903)
Race				
American Indian or Alaska Native	0.0% (0)	0.3% (1)	0.0% (0)	0.1% (1)
Asian	0.8% (1)	1.1% (4)	3.1% (16)	2.1% (21)
Black or African American	4.0% (5)	17.2% (61)	9.3% (48)	11.4% (114)
Native Hawaiian or other Pacific Islander	0.0% (0)	0.3% (1)	0.0% (0)	0.1% (1)
White	93.6% (117)	80.0% (284)	86.9% (450)	85.3% (851)
Other or multiple	1.6% (2)	1.1% (4)	0.8% (4)	1.0% (10)
BMI (kg/m^2^)				
N	124	354	518	996
Mean (SD)	29.9 (5.4)	30.5 ± 5.6	30.3 ± 5.9	30.3 ± 5.7
CV-related history				
Congestive heart failure	6.4% (8)	16.3% (58)	12.4% (64)	13.0% (130)
Cerebrovascular accident	7.2% (9)	8.2% (29)	9.3% (48)	8.6% (86)
Transient ischemic attack	6.4% (8)	8.2% (29)	7.9% (41)	7.8% (78)
Peripheral arterial disease	8.8% (11)	16.6% (59)	14.1% (73)	14.3% (143)
Myocardial infarction	31.2% (39)	38.0% (135)	34.0% (176)	35.1% (350)
Coronary artery disease	93.6% (117)	81.1% (288)	83.0% (430)	83.7% (835)
Type II diabetes mellitus	21.6% (27)	37.2% (132)	37.1% (192)	35.2% (351)
Family history of premature ASCVD	35.2% (44)	37.5% (133)	40.9% (212)	39.0% (389)
LDL-C <70 mg/dL, % (n/N)	60.6% (40/66)	29.1% (52/179)	39.9% (109/273)	38.8% (201/518)
Lipids, median (Q1, Q3), mg/dL^b^^,^^c^				
LDL-C	n = 66	n = 179	n = 273	N = 518
	62.0 (40.0, 90.0)	89.0 (64.0, 113.0)	74.0 (60.0, 89.0)	77.5 (60.0, 98.0)
HDL-C	n = 65	n = 178	n = 272	N = 515
	48.0 (42.0, 60.0)	49.0 (39.0, 58.0)	45.0 (38.0, 53.5)	46.0 (39.0, 57.0)
Triglycerides	n = 65	n = 177	n = 270	N = 512
	120.0 (87.0, 146.0)	118.0 (88.0, 170.0)	109.5 (81.0, 154.0)	111.5 (84.0, 159.0)
Total cholesterol	n = 65	n = 179	n = 272	N = 516
	146.0 (117.0, 172.0)	160.0 (133.0, 198.0)	143.0 (127.0, 163.0)	150.0 (127.5, 176.0)

Values are % (n) unless otherwise indicated.

ASCVD = atherosclerotic cardiovascular disease; BMI = body mass index; CV = cardiovascular; EDU = Education; GOULD = Getting to an Improved Understanding of Low-Density Lipoprotein Cholesterol and Dyslipidemia Management; HDL-C = high-density lipoprotein cholesterol; LDL-C = low-density lipoprotein cholesterol; mAb = monoclonal antibody; PCSK9i = proprotein convertase subtilisin/kexin type 9 inhibitor.

a Baseline data were from the last chart review between July 1, 2019, and the start of GOULD EDU (June 30, 2020).

b If no lipid value was in the chart between July 1, 2019, and June 30, 2020, data were considered missing.

c For LDL-C, HDL-C and total cholesterol, 1 mg/dL = 1/38.6 mmol/L; for triglycerides, 1 mg/dL = 1/88.5 mmol/L.

### LLT at baseline

At the start of GOULD EDU, the percentages of patients prescribed statins in each of the GOULD baseline LDL-C cohorts were as follows: 82.0% (291/355) of the LDL-C ≥100 mg/dL cohort and 94.2% (488/518) of the LDL-C 70 to 99 mg/dL cohort. High intensity statins were used in 42.8% (152/355) of the LDL-C ≥100 mg/dL cohort and 52.3% (271/518) of the LDL-C 70 to 99 mg/dL cohort. Ezetimibe was prescribed for 15.8% (56/355) of the LDL-C ≥100 mg/dL cohort and 11.4% (59/518) of the LDL-C 70 to 99 mg/dL cohort. PCSK9i monoclonal antibody (mAb) treatment was prescribed in 10.4% (37/355) of the LDL-C ≥100 mg/dL cohort and 3.1% (16/518) of the LDL-C 70 to 99 mg/dL cohort.

Of patients in the PCSK9i mAb cohort at the start of GOULD, 91.2% (114/125) were on a PCSK9i at the start of GOULD EDU; 42.4% (53/125) were taking PCSK9i as monotherapy, and the remainder combined PCSK9i treatment with statin (20.0%, 25/125), ezetimibe (11.2%, 14/125), both statin and ezetimibe (5.6%, 7/125), or another LLT (12.0%, 15/125).

### Changes in LLT intensity

**Figure 1:**
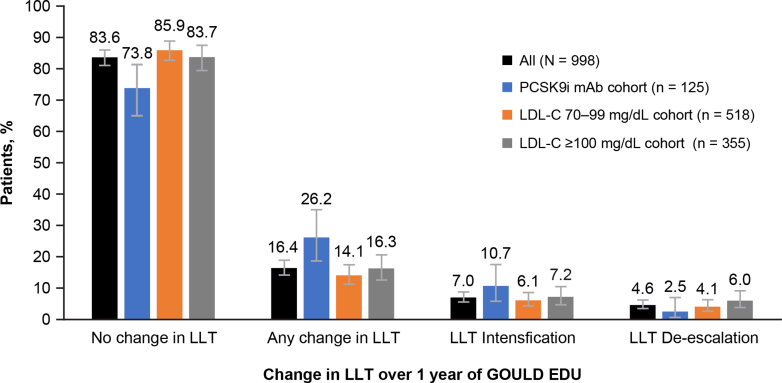
**Changes in LLT Intensity During GOULD EDU** EDU = Education; GOULD = Getting to an Improved Understanding of Low-Density Lipoprotein Cholesterol and Dyslipidemia Management; LDL-C = low-density lipoprotein cholesterol; LLT = lipid-lowering therapy; mAb = monoclonal antibody; PCSK9i = proprotein convertase subtilisin/kexin type 9 inhibitor.

**Figure 2:**
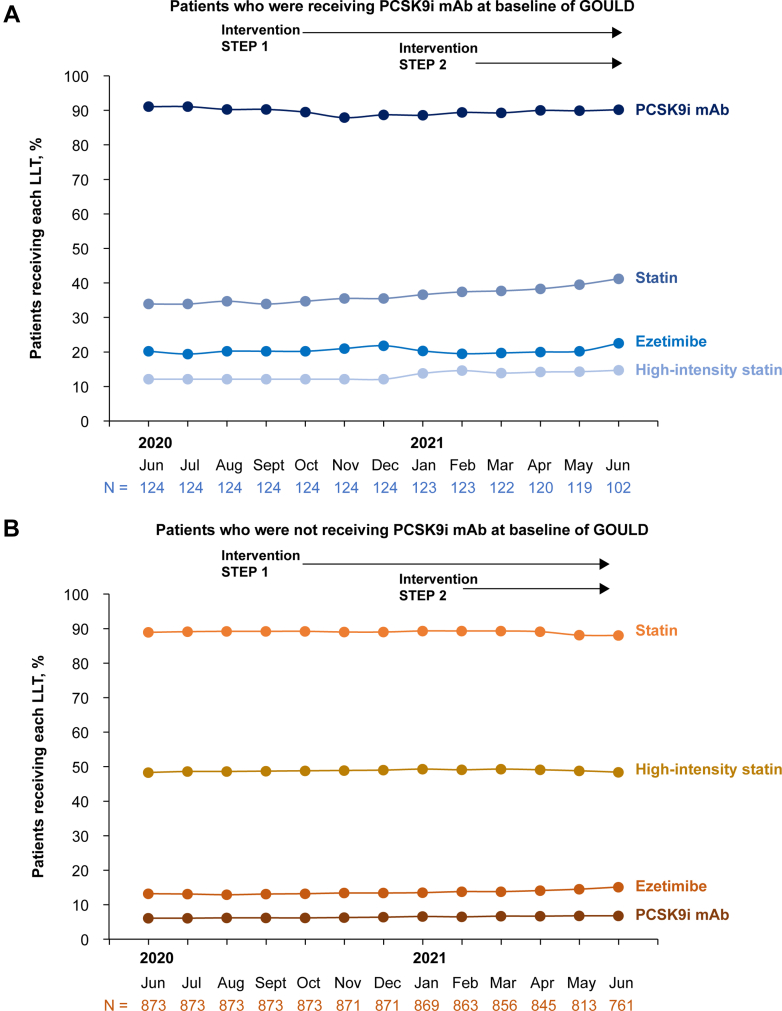
**LLTs Received Over Time in GOULD EDU** (A) Patients who were receiving PCSK9i therapy. (B) Patients who were not receiving PCSK9i therapy at baseline of the GOULD parent study. Note: Figure B combines patients who were in the LDL-C ≥100 mg/dL cohort and the LDL-C 70 to 99 mg/dL cohort at GOULD baseline. Abbreviations as in Figure 1.

Intensification of LLT occurred in only 7.0% (69/981) of patients during the 1-year GOULD EDU extension period. Overall, minimal change occurred in LLT (Figures 1 and 2, Supplemental Table 3), with 83.6% (820/981) of patients having no change in LLT. De-escalation occurred in 4.6% (45/981) of patients and change in therapy without changing intensity (eg, switching to a statin of similar intensity) occurred in 4.8% (47/981) of patients. Similar patterns of minimal LLT intensification occurred in those randomized to Steps 1 vs 2 (Supplemental Table 4, Supplemental Figure 4) and in Period 1 vs 2 of the study (data not shown). Pearson chi-square tests comparing for any change in LLT across Steps 1 vs 2 show no significant differences (Supplemental Table 4).

Statins were up-titrated in 1.8% (18/981) of patients, down-titrated in 0.9% (9/981), and discontinued in 2.4% (24/981). Ezetimibe was added in 2.1% (21/981) of patients and discontinued in 0.6% (6/981) of patients. Overall, PCSK9i was added in 0.9% (9/981) of patients and discontinued in 0.7% (7/981) of patients. Fish oil was added in 0.9% (9/981) of patients and discontinued in 1.7% (17/981) of patients. Other lipid-lowering medications (see Supplemental Table 4) were added in 0.9% (9/981) of patients and discontinued in 0.8% (8/981) of patients.

Patients in the PCSK9i cohort had a slightly greater percentage of patients with changes in therapy than the LDL-C cohorts; 26.2% (32/122) had some change to LLT, including intensification in 13 patients, de-escalation in 3 patients, and other changes that did not affect intensity in 16 patients. Of patients in the PCSK9i cohort at the beginning of GOULD EDU, 2.5% (3/122) discontinued PCSK9i therapy during GOULD EDU.

### LDL-C goal achievement

**Figure 3:**
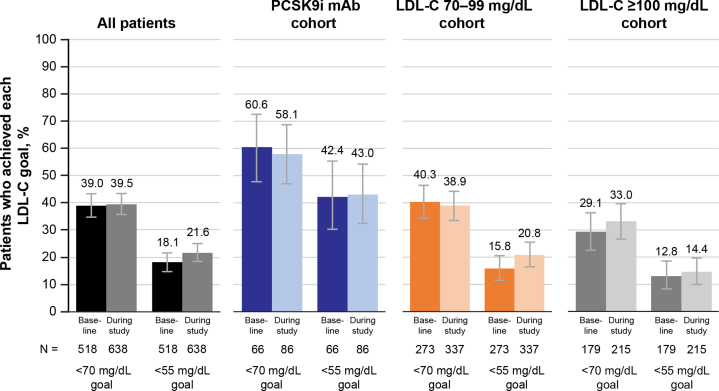
**Patients Achieving LDL-C Goals of <70 mg/dL and <55 mg/dL During GOULD EDU** LDL-C values are the last recorded value between July 1, 2020, and the end of study. If no value was recorded during this period, either due to incomplete chart review or lipid panels not being ordered, the value was considered missing. LDL-C conversion: 1 mg/dL = 1/38.6 mmol/L. Abbreviations as in Figure 1.

Figure 3 shows the percentage of patients meeting the goal of LDL-C <70 mg/dL or <55 mg/dL during GOULD EDU. At the start of GOULD EDU, of patients who had LDL-C measured in the previous year, 39.0% (202/518) had LDL-C <70 mg/dL and 18.1% (94/518) had LDL-C <55 mg/dL. Over the course of GOULD EDU, little change occurred (Central Illustration), with 39.5% (252/638) of patients reaching LDL-C <70 and 21.6% (138/638) reaching LDL-C <55. A greater percentage of patients achieved these goals during GOULD EDU in the PCSK9i cohort (LDL-C <70 mg/dL: 58.1% [50/86]; LDL-C <55 mg/dL: 43.0% [37/86]) than in the LDL-C 70 to 99 cohort (38.9% [131/337]; 20.8% [70/337]) or the LDL-C >100 cohort (33.0% [71/215]; 14.4% [31/215]); however, little improvement occurred compared with baseline for any of the cohorts.

**Central Illustration:**
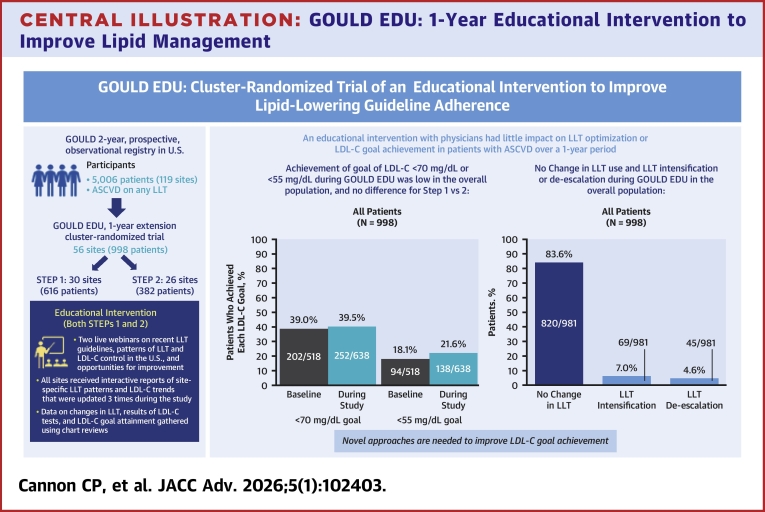
**GOULD EDU: 1-Year Educational Intervention to Improve Lipid Management** The objective was to determine whether an educational intervention targeting physicians improves optimization of LLT over 1 year for patients with ASCVD. The results showed that an educational intervention with physicians had little impact on LLT optimization or LDL-C goal achievement in patients with ASCVD over a 1-year period. Abbreviations: ASCVD = atherosclerotic cardiovascular disease; other abbreviations as in Figure 1.

### Change in median LDL-C during GOULD EDU

**Figure 4:**
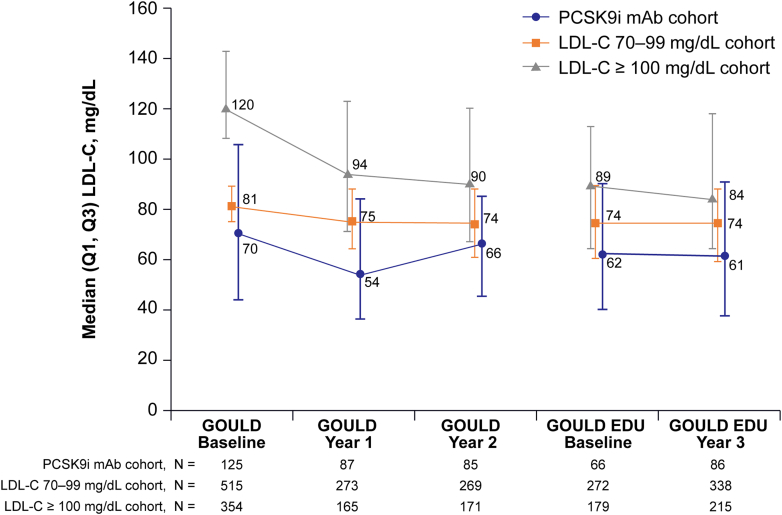
**Median LDL-C During GOULD and GOULD EDU** Figure includes patients during 3 years of follow-up in both GOULD and GOULD EDU. Note: For GOULD EDU baseline values, LDL-C is the last recorded value between July 1, 2019, and June 30, 2020. For GOULD EDU year 3 values, LDL-C is the last recorded value between July 1, 2020, and the end of study. If no value was recorded for a designated study period, either due to incomplete chart review or lipid panels not being ordered, the value was considered missing. Abbreviations as in Figure 1.

Although some reduction in LDL-C occurred during GOULD, particularly in the first year of the study, minimal change in LDL-C occurred during GOULD EDU (Figure 4). Of those with LDL-C measured, almost no change occurred in median (Q1, Q3) LDL-C in the PCSK9i cohort (62 mg/dL [40, 90] to 61 mg/dL [37, 91]) or the LDL-C ≥70 to 99 mg/dL cohort (74 mg/dL [60, 89] to 74 mg/dL [59, 88]). The LDL-C >100 mg/dL cohort had a modest reduction from 89 mg/dL (64, 113) to 84 mg/dL (64, 118). Similarly, minimal changes in LDL-C occurred during GOULD EDU when looking at Step 1 and Step 2 individually (Supplemental Figures 5A and 5B). When the analysis was repeated with last observation carried forward for missing LDL-C values, the data were very similar (Supplemental Figure 5C). Comparing Steps 1 vs 2, we see that there is no significant difference in changes in LDL-C between the 2 steps (Wilcoxon rank sum test *P* = 0.49). A similar test using the last observation carried forward approach also shows no significant difference between the 2 steps (Wilcoxon rank sum test *P* = 0.23).

### Frequency of lipid panels

Almost half of patients (48.1%, 480/998) did not have an LDL-C value recorded in their chart in the 1-year period before GOULD EDU began. During the 1-year period of GOULD EDU, 66.0% (659/998) of patients had at least one lipid panel; 34.0% (339/998) of patients had no lipid panels, 43.3% (432/998) had one panel, 21.1% (211/998) had 2 panels, and 1.6% (16/998) had more than 2 panels. Patients in the PCSK9i mAb cohort had more lipid panels and had fewer days between their latest lipid panels than the other cohorts (median [Q1, Q3] of 308 [180, 521] days for the PCSK9i cohort vs 416 [202, 613] for the LDL-C ≥100 mg/dL cohort and 365 [205, 579] days for the LDL-C 70 to 99 mg/dL cohort).

### Responses from physician questionnaire

The educational intervention had little impact on the questionnaire responses from physicians (N = 49), including items regarding LLT guidelines, appropriate LDL-C goals and testing frequency, or when to intensify treatment (Table 2, Supplemental Table 5).

**Table 2:** Physician Questionnaire Responses to Selected Items Before and After the Educational Intervention

	Before Educational Intervention (n = 49)	After Educational Intervention (n = 49)
What is the LDL-C goal that you aim to achieve with cholesterol-lowering therapy for your patients with ASCVD?		
Less than 50 mg/dL	12.2% (6/49)	18.4% (9/49)
Less than 70 mg/dL	69.4% (34/49)	65.3% (32/49)
Less than 100 mg/dL	8.2% (4/49)	4.1% (2/49)
Less than 130 mg/dL	0.0% (0/49)	0.0% (0/49)
It depends on patient’s other risk factors	10.2% (5/49)	8.2% (4/49)
Do not use LDL-C goals	0.0% (0/49)	4.1% (2/49)
What do you usually do when a patient has very low LDL cholesterol—for example, <25 mg/dL? (for patients with ASCVD)		
Make no change to LLT	55.1% (27/49)	63.3% (31/49)
Reduce the lipid-lowering medication intensity	38.8% (19/49)	20.4% (10/49)
Discontinue one or more lipid-lowering medication	6.1% (3/49)	10.2% (5/49)
Switch lipid-lowering drugs	0.0% (0/49)	2.0% (1/49)
Other	0.0% (0/49)	4.1% (2/49)
After a patient with ASCVD first initiates statins, how long do you wait until you first reassess their lipid levels?		
Less than 3 months	42.9% (21/49)	51.0% (25/49)
Three to 6 months	53.1% (26/49)	40.8% (20/49)
6 months to 1 year	2.0% (1/49)	8.2% (4/49)
More than 1 year	0.0% (0/49)	0.0% (0/49)
Never	2.0% (1/49)	0.0% (0/49)
For patients with ASCVD taking a maximally tolerated statin dose having high LDL-C measurement…how frequently do you consider adding a nonstatin lipid-lowering drug?		
Never	0.0% (0/49)	0.0% (0/49)
Rarely	0.0% (0/49)	6.1% (3/49)
Some of the time	53.1% (26/49)	53.1% (26/49)
Most of the time	30.6% (15/49)	18.4% (9/49)
Always	16.3% (8/49)	22.4% (11/49)
In your opinion, how strong is the scientific evidence supporting the use of high-intensity statins vs low-/moderate-intensity statins among patients with ASCVD?		
Very strong	44.9% (22/49)	44.9% (22/49)
Strong	38.8% (19/49)	36.7% (18/49)
Moderate	16.3% (8/49)	12.2% (6/49)
Weak	0.0% (0/49)	4.1% (2/49)
Very weak	0.0% (0/49)	0.0% (0/49)
Missing	0.0% (0/49)	2.0% (1/49)
Effect of GOULD results on my practice…Would you make any changes in your practice based on the data you see?		
No changes to treatment	30.6% (15/49)	40.8% (20/49)
Treat LDL-C more aggressively and aim to get ASCVD patients to LDL <70	40.8% (20/49)	26.5% (13/49)
Aim to treat very high-risk ASCVD patients to an LDL-C threshold <55 mg/dL	26.5% (13/49)	28.6% (14/49)
Not target LDL levels, but aim to increase use of intensive statin therapy	2.0% (1/49)	4.1% (2/49)
In your patients who are nonadherent to their statins, what is the most common reason for nonadherence?		
Patients don't believe the medication works	2.0% (1/49)	0.0% (0/49)
Patients don't have access to a pharmacy	0.0% (0/49)	0.0% (0/49)
Patients can't afford the medication	10.2% (5/49)	4.1% (2/49)
Patients experienced side effects	79.6% (39/49)	81.6% (40/49)
Patients don't like taking medications	4.1% (2/49)	14.3% (7/49)
Other	4.1% (2/49)	0.0% (0/49)

Values are n/N (%).

LLT = lipid-lowering therapy; other abbreviations as in Table 1.

## Discussion

Underutilization of LLTs and poor LDL-C goal attainment were reported in the first 2 years of GOULD,^4^ and similar data have been reported in many other studies of patients with ASCVD.^9^ We followed patients for an additional year that included a physician-targeted educational intervention in GOULD EDU, and only a small number of patients had LLT intensification, despite 61% (316/518) having LDL-C >70 mg/dL at baseline. Thus, the educational intervention and access to an Interactive GOULD Registry Site Report were not effective in helping promote guideline adherence in prescribing LLT among either cardiologists or other physicians.

Our physician survey results show that most physicians considered <70 mg/dL to be their LDL-C goal and that LDL-C testing should be done yearly. However, only 39.5% (252/638) of patients achieved LDL-C below 70 mg/dL, and only 66% (659/998) of patients had their LDL-C tested during the 1-year period. The more stringent goal of LDL-C <55 mg/dL was attained by only 18% (101/552) of the LDL-C cohorts, and 43% (37/86) of the PCSK9i cohort. Despite not reaching LDL-C goals, a PCSK9i was added in <1% (9/981) of patients in GOULD EDU, which is consistent with findings in the prior 2 years of GOULD.

The low intensification rates and low rates of LDL-C goal achievement seen in the GOULD study expose patients to unnecessary risk of CV events, which accelerates over time with cumulative LDL-C exposure. Potent reduction of LDL-C to low levels (ie, <55 mg/dL) is associated with atherosclerosis regression and plaque stabilization,^10^, ^11^, ^12^, ^13^ and the benefit of LLT on ASCVD event reduction is proportional to the decrease in absolute LDL-C^14^ and the level and duration of low LDL-C achieved.^15^ Even in this selected group of physicians participating in a study of LLT, some reported reducing intensity of LLT because LDL-C was considered too low. However, greater risk reduction is achieved at very low levels of LDL-C, and no safety issues have been identified, even at levels < 25 mg/dL.^16^^,^^17^

The reasons for low rates of LLT intensification in GOULD and other studies of real-world clinical practice are likely complex,^9^^,^^18^^,^^19^ and many other intervention studies have also failed to improve rates of initiation and intensification of LLTs in various patient groups. Barriers to guideline implementation are multifaceted and occur at multiple levels, including patient, physician, health system, health plan, and pharmacy levels.^18^ Our intervention focused on the physician level and emphasized education through multiple avenues but was not effective. Some have suggested a Dunning-Kruger effect might be part of the explanation. Multifaceted intervention strategies that use a team approach, have systemic or institutional support, and address multiple barriers simultaneously may be most effective in changing clinical practice and improving outcomes.^20^, ^21^, ^22^ Parsing out where education does and does not lead to improvement may help in further educational endeavors. For example, the comprehensive, coordinated care model used by the Cardiometabolic Center Alliance has recently shown improvements in use of guideline-directed therapies for cardiometabolic diseases with accompanying reductions in patients’ CV risk factors, including lipid levels.^23^ The use of practical supports embedded in clinical practice, such as electronic reminders about guidelines and alerts in the electronic medical record when patients are above target LDL-C levels, have been shown to be effective in recent studies of lipid lowering,^19^ antithrombotic therapy,^24^ diabetes,^25^ and heart failure.^26^ To achieve substantial improvement in LLT guideline adherence, quality metrics for LDL-C monitoring, similar to those used for blood pressure and diabetes, are likely to be needed.^27^

### Study Limitations

The study has some limitations. The study was conducted in the United States only and patient adherence to LLT was not monitored. A limitation of our intervention is that it provided practice-specific data to the principal investigator (PI) physician at each site, and although the study provided support, it was incumbent on the PI physician to distribute the information and report to the other providers at their site; however, a clinician-specific report may have had greater impact on individual physician behavior. In addition, the educational intervention may not have produced sufficient engagement with the educational material. Change in knowledge is a foundation for change in behavior, but real-time reminders, alerts, and other supports and incentives may be needed for measurable change to clinical practice. Our survey was also only completed by the site PI, who managed some patients at their site, but we do not have full survey data of all treating physicians. Finally, the study primarily focuses on complete-case analyses where missing data are ignored, which can potentially bias the results if data are missing not at random. Supplemental Figure 3 provides an overview of subjects that completed chart review.

## Conclusions

A 1-year educational intervention directed at physicians led to almost no improvement in LLT intensification in patients with ASCVD, of whom only 39.5% had LDL-C <70 mg/dL and only 21.6% had LDL-C <55 mg/dL. More intensive interventions, such as changes in clinical care delivery models and reinstatement of LDL-C quality metrics, may be required to address clinical inertia regarding LLT optimization and the complex reasons for suboptimal adherence to guidelines.

## Funding support and author disclosures

This study was funded by 10.13039/100002429Amgen (Thousand Oaks, CA). Dr Cannon has received research grants from 10.13039/100002429Amgen, Better Therapeutics, 10.13039/100001003Boehringer Ingelheim (BI), 10.13039/501100004191Novo Nordisk, and salary support from Colorado Prevention Center (CPC) Clinical Research, which gets research grant support from 10.13039/100002429Amgen, 10.13039/100004326Bayer, Cleerly, 10.13039/501100022336Esperion, Lexicon, and Silence; has received consulting fees from Amryt/Chiesi, 10.13039/100002429Amgen, Ascendia, 10.13039/100005614Biogen, BI, 10.13039/100002491BMS, 10.13039/100008322CSL Behring, Genomadix, Lilly, 10.13039/100005565Janssen, Lexicon, Milestone, 10.13039/100004336Novartis, 10.13039/100004319Pfizer, and Rhoshan; and has served on data and safety monitoring boards for the Areteia, 10.13039/501100004191Novo Nordisk, ROMTherapy, Inc, and the Veterans Administration. Dr Ballantyne has received research grants (all paid to institution) from 10.13039/100014386Abbott Diagnostics, Akcea, 10.13039/100002429Amgen, 10.13039/501100022336Arrowhead, Eli Lilly, Ionis, Merck, 10.13039/100004336Novartis, 10.13039/100009857Novo Nordisk, 10.13039/100016545Roche Diagnostic, 10.13039/100000002NIH, 10.13039/100000968American Heart Association, and 10.13039/100000041ADA; and has received consulting income from 89Bio, 10.13039/100014386Abbott Diagnostics, 10.13039/100002429Amgen, Arrowhead, 10.13039/100004325AstraZeneca, Denka Seiken, 10.13039/501100022336Esperion, Genentech, HeartFlow, Ionis, Eli Lilly, 10.13039/100004334Merck, New Amsterdam, 10.13039/100004336Novartis, 10.13039/100009857Novo Nordisk, and 10.13039/100016545Roche Diagnostic. Dr Bhatt is on the Advisory Board for Angiowave, Antlia Bioscience, 10.13039/100004326Bayer, 10.13039/100001003Boehringer Ingelheim, CellProthera, Cereno Scientific, E-Star Biotech, High Enroll, 10.13039/100008897Janssen, Level Ex, McKinsey, Medscape Cardiology, 10.13039/100004334Merck, NirvaMed, 10.13039/501100004191Novo Nordisk, Repair Biotechnologies, Stasys, SandboxAQ (stock options), Tourmaline Bio; is on the Board of Directors of American Heart Association New York City, Angiowave (stock options), Bristol Myers Squibb (stock), DRS.LINQ (stock options), High Enroll (stock); is a consultant for Alnylam, Altimmune, Broadview Ventures, Corcept Therapeutics, Corsera, GlaxoSmithKline, Hims, SERB, SFJ, Summa Therapeutics, and Worldwide Clinical Trials; is on the Data Monitoring Committees of Acesion Pharma, Assistance Publique-Hôpitaux de Paris, Baim Institute for Clinical Research, Boston Scientific (Chair, PEITHO trial), Cleveland Clinic, Contego Medical (Chair, PERFORMANCE 2), Duke Clinical Research Institute, Mayo Clinic, Mount Sinai School of Medicine (for the ABILITY-DM trial, funded by Concept Medical; for ALLAY-HF, funded by Alleviant Medical), 10.13039/100004336Novartis, Population Health Research Institute; Rutgers University (for the NIH-funded MINT Trial); has received honoraria from American College of Cardiology (Senior Associate Editor, Clinical Trials and News, American College of Cardiology.org; Chair, American College of Cardiology Accreditation Oversight Committee), Arnold and Porter law firm (work related to Sanofi/Bristol-Myers Squibb clopidogrel litigation), Baim Institute for Clinical Research (AEGIS-II executive committee funded by CSL Behring), Belvoir Publications (Editor in Chief, Harvard Heart Letter), Canadian Medical and Surgical Knowledge Translation Research Group (clinical trial steering committees), CSL Behring (American Heart Association lecture), Duke Clinical Research Institute, Engage Health Media, HMP Global (Editor in Chief, *Journal of Invasive Cardiology*), Medtelligence/ReachMD (CME steering committees), MJH Life Sciences, Oakstone CME (Course Director, Comprehensive Review of Interventional Cardiology), Philips (Becker's Webinar on AI), Population Health Research Institute, WebMD (CME steering committees), Wiley (steering committee); Other: Clinical Cardiology (Deputy Editor); Progress in Cardiovascular Diseases (Deputy Editor); has a Patent for Sotagliflozin (named on a patent for sotagliflozin assigned to Brigham and Women's Hospital who assigned to Lexicon; neither I nor Brigham and Women's Hospital receive any income from this patent); has received research funding from Abbott, Acesion Pharma, Afimmune, Alnylam, Amarin, 10.13039/100002429Amgen, 10.13039/100004325AstraZeneca, Atricure, 10.13039/100004326Bayer, 10.13039/100001003Boehringer Ingelheim, Boston Scientific, CellProthera, Cereno Scientific, Chiesi, Cleerly, CSL Behring, Faraday Pharmaceuticals, Fractyl, Idorsia, 10.13039/100008897Janssen, Javelin, Lexicon, Lilly, Medtronic, 10.13039/100004334Merck, MiRUS, Moderna, 10.13039/100004336Novartis, 10.13039/501100004191Novo Nordisk, Pfizer, PhaseBio, 10.13039/100009857Regeneron, Reid Hoffman Foundation, Roche, Sanofi, Stasys, and 89Bio; has received royalties from Elsevier (Editor, Braunwald’s Heart Disease); is a Site Co-Investigator from Cleerly. Dr de Lemos has received consulting income from participation in DSMBs from 10.13039/100002429Amgen, 10.13039/100009857Regeneron, 10.13039/100008897Janssen, Eli Lilly, 10.13039/100004334Merck, 10.13039/501100004191Novo Nordisk, and Verve Therapeutics. Dr Rosenson has received personal fees from 10.13039/100002429Amgen, Arrowhead, CRISPR Therapeutics, Eli Lilly, 10.13039/100004336Novartis, Precision Biosciences, and 10.13039/100009857Regeneron during the conduct of the study and personal fees from the Amgen Steering Committee outside the submitted work. Drs Mues and Exter are former Amgen employees and own Amgen stock. Dr Alam is an Amgen employee and owns Amgen stock. Dr Kosiborod has received research grants from 10.13039/100004325AstraZeneca, 10.13039/100001003Boehringer Ingelheim; other research support from 10.13039/100004325AstraZeneca; is on the consulting/advisory boards for Alnylam, 10.13039/100002429Amgen, Applied Therapeutics, 10.13039/100004325AstraZeneca, 10.13039/100004326Bayer, 10.13039/100001003Boehringer Ingelheim, Eli Lilly, Esperion Therapeutics, 10.13039/100008897Janssen, Lexicon, 10.13039/100004334Merck (Diabetes and Cardiovascular), 10.13039/501100004191Novo Nordisk, Pharmacosmos, Sanofi, and Vifor Pharma; has received honoraria from 10.13039/100004325AstraZeneca, 10.13039/100001003Boehringer Ingelheim, and 10.13039/501100004191Novo Nordisk; and is currently an employee of and owns stock in 10.13039/100004325AstraZeneca. All other authors have reported that they have no relationships relevant to the contents of this paper to disclose. Researchers from the sponsor were involved in the design and conduct of the study; they, along with the study co-chairs, oversaw the data coordinating center and managed data collection. They were not involved in performing the data analysis, which was done by the statistical center (Baim Institute for Clinical Research) but did review and provide comments on the data analyses and the manuscript. The decision to submit the manuscript for publication was by the study co-chairs.
