# Supplementary material for: GOULD EDU: Cluster-Randomized Trial of an Educational Intervention to Improve Lipid-Lowering Guideline Adherence
Source: JACC Adv. 2025 Dec 3;5(1):102403. doi: 10.1016/j.jacadv.2025.102403 (PMC12721036; doi:10.1016/j.jacadv.2025.102403)
Supplement: Supplemental Material [file mmc1.docx]

**SUPPLEMENTAL APPENDIX**

**GOULD EDU: Cluster-Randomized Trial of an Educational Intervention to Improve Lipid-Lowering Guideline Adherence**

Christopher P. Cannon, MD; Christie M. Ballantyne, MD; Deepak L. Bhatt, MD, MPH, MBA; James A. de Lemos, MD; Qi Gao, MS; Naishu Kui, MS; Robert S. Rosenson, MD; Katherine E. Mues, PhD, MPH; Jason Exter, PharmD; Shushama Alam, PharmD; Mikhail N. Kosiborod, MD; on behalf of the GOULD-EDU Investigators

| **Element** | **Page** |
| --- | --- |
| Supplemental Table 1. Demographic and Disease Characteristics at Baseline of GOULD EDU for Patients in Step 1 and Step 2 | 2 |
| Supplemental Table 2. Demographic and Disease Characteristics at Baseline of GOULD for Patients Who Did or Did Not Enroll in GOULD EDU | 3 |
| Supplemental Table 3. Changes in LLT During GOULD EDU by Cohort From the GOULD Parent Study | 4 |
| Supplemental Table 4. Changes in LLT during GOULD EDU by Intervention Step and Cohort From the GOULD Parent Study | 5 |
| Supplemental Table 5. Physician Questionnaire Responses   1. Clinical guidelines 2. LDL-C goals and low LDL-C 3. Lipid measurement during statin treatment 4. Treating ASCVD patients with maximally tolerated statin therapy who have high LDL-C measurement 5. Statin therapy evidence and side effects 6. Frequency of prescribing non-statin LLT 7. PCSK9 inhibitor monoclonal antibody treatments 8. Familial hypercholesterolemia 9. Patient education 10. Treatment targets 11. Effect of GOULD results on physician practice | 8 |
| Supplemental Figure 1. Study Design of GOULD and GOULD EDU | 19 |
| Supplemental Figure 2. Sample Figures From Interactive GOULD Registry Site Report | 20 |
| Supplemental Figure 3. Patient Flow Diagram | 23 |
| Supplemental Figure 4. Percentages of Patients With Changes or No Changes in Lipid-Lowering Therapy Intensity During GOULD EDU for Sites Randomized to Step 1 (a) or Step 2 (b) | 24 |

**Supplemental Table 1. Demographic and Disease Characteristics at Baseline of GOULD EDU for Patients in Step 1 and Step 2**

| **Baseline Characteristic^a,b^** | **Step 1 of GOULD EDU (N = 616)** | **Step 2 of GOULD EDU**  **(N=382)** |
| --- | --- | --- |
| Age (years), mean (SD) | 70.1 (9.9) | 70.4 (9.2) |
| Male | 61.0% (376/616) | 61.3% (234/382) |
| Ethnicity |  |  |
| Hispanic or Latino | 11.0% (68/616) | 6.5% (25/382) |
| Not Hispanic or Latino | 89.0% (548/616) | 92.9% (355/382) |
| Race |  |  |
| American Indian or Alaska Native | 0.0% (0/616) | 0.3% (1/382) |
| Asian | 3.1% (19/616) | 0.5% (2/382) |
| Black or African American | 15.4% (95/616) | 5.0% (19/382) |
| Native Hawaiian or Other Pacific Islander | 0.2% (1/616) | 0.0% (0/382) |
| White | 80.0% (493/616) | 93.7% (358/382) |
| Other or multiple | 1.3% (8/616) | 0.5% (2/382) |
| BMI (kg/m^2^) |  |  |
| N | 615 | 381 |
| Mean (SD) | 30.4±5.9 | 30.2±5.4 |
| CV-related history |  |  |
| Congestive heart failure | 13.0% (80/616) | 13.1% (50/382) |
| Cerebrovascular accident | 8.6% (53/616) | 8.6% (33/382) |
| Transient ischemic attack | 6.0% (37/616) | 10.7% (41/382) |
| Peripheral arterial disease | 15.6% (96/616) | 12.3% (47/382) |
| Myocardial infarction | 32.5% (200/616) | 39.3% (150/382) |
| Coronary artery disease | 83.4% (514/616) | 84.0% (321/382) |
| Type II diabetes mellitus | 33.4% (206/616) | 38.0% (145/382) |
| Family history of premature ASCVD | 39.4% (243/616) | 38.2% (146/382) |

^a^Baseline data were from the last chart review between July 1, 2019, and the start of GOULD EDU (June 30, 2020).

^b^Values are shown as percentage (n), unless otherwise indicated.

Abbreviations: ASCVD, atherosclerotic cardiovascular disease; BMI, body mass index; CV, cardiovascular; SD, standard deviation.

**Supplemental Table 2. Demographic and Disease Characteristics at Baseline of GOULD for Patients Who Did or Did Not Enroll in GOULD EDU**

| **Baseline Characteristic^a,b^** | **Participated in  GOULD EDU (N = 998)** | **Did not participate in GOULD EDU**  **(N=3918)** |
| --- | --- | --- |
| Age (years), mean (SD) | 67.5 (9.6) | 67.9 (10.0) |
| Male | 61.1% (610/998) | 60.3% (2364/3918) |
| Ethnicity |  |  |
| Hispanic or Latino | 9.3% (93/998) | 7.3% (287/3918) |
| Not Hispanic or Latino | 90.5% (903/998) | 91.9% (3602/3918) |
| Race |  |  |
| American Indian or Alaska Native | 0.1% (1/998) | 0.2% (6/3918) |
| Asian | 2.1% (21/998) | 1.7% (67/3918) |
| Black or African American | 11.4% (114/998) | 9.4% (367/3918) |
| Native Hawaiian or Other Pacific Islander | 0.1% (1/998) | 0.1% (5/3918) |
| White | 85.3% (851/998) | 86.8% (3399/3918) |
| Other or multiple | 1.0% (10/998) | 1.9% (74/3918) |
| BMI (kg/m^2^) |  |  |
| N | 996 | 3872 |
| Mean (SD) | 30.3 (5.7) | 30.7 (6.2) |
| CV-related history |  |  |
| Congestive heart failure | 12.2% (122/998) | 11.9% (468/3918) |
| Cerebrovascular accident | 8.1% (81/998) | 10.8% (425/3918) |
| Transient ischemic attack | 7.0% (70/998) | 8.7% (339/3918) |
| Peripheral arterial disease | 14.3% (143/998) | 13.6% (532/3918) |
| Myocardial infarction | 33.9% (338/998) | 31.5% (1233/3918) |
| Coronary artery disease | 83.7% (835/998) | 79.8% (3126/3918) |
| Type II diabetes mellitus | 35.2% (351/998) | 33.5% (1314/3918) |
| Family history of premature ASCVD | 39.0% (389/998) | 33.8% (1326/3918) |

^a^Baseline data were from the baseline chart review from the GOULD parent study.

^b^Values are shown as percentage (n), unless otherwise indicated.

Abbreviations: ASCVD, atherosclerotic cardiovascular disease; BMI, body mass index; CV, cardiovascular; SD, standard deviation.

**Supplemental Table 3.** **Changes in LLT During GOULD EDU by Cohort From the GOULD Parent Study**

| **LLT change, % (n/N)** | **Cohort assigned at Baseline of  GOULD Parent Study** | | | **Total (N = 998)** |
| --- | --- | --- | --- | --- |
|  | **PCSK9i mAb (n = 125)** | **No PCSK9i Treatment** | |  |
|  |  | **LDL-C ≥ 100 mg/dL  (n = 355)** | **LDL-C 70–99  mg/dL (n = 518)** |  |
| No change in LLT | 73.8% (90/122) | 83.7% (292/349) | 85.9% (438/510) | 83.6% (820/981) |
| Any change in LLT | 26.2% (32/122) | 16.3% (57/349) | 14.1% (72/510) | 16.4% (161/981) |
| Lipid treatment intensification | 10.7% (13/122) | 7.2% (25/349) | 6.1% (31/510) | 7.0% (69/981) |
| Statin up-titrated | 0.8% (1/122) | 1.1% (4/349) | 2.5% (13/510) | 1.8% (18/981) |
| Statin added | 5.7% (7/122) | 1.7% (6/349) | 1.2% (6/510) | 1.9% (19/981) |
| Ezetimibe added | 2.5% (3/122) | 2.0% (7/349) | 2.2% (11/510) | 2.1% (21/981) |
| PCSK9i added | 1.6% (2/122) | 1.4% (5/349) | 0.4% (2/510) | 0.9% (9/981) |
| Lipid treatment de‑escalation | 2.5% (3/122) | 6.0% (21/349) | 4.1% (21/510) | 4.6% (45/981) |
| Statin down-titrated | 0.0% (0/122) | 1.1% (4/349) | 1.0% (5/510) | 0.9% (9/981) |
| Statin discontinued | 0.8% (1/122) | 3.2% (11/349) | 2.4% (12/510) | 2.4% (24/981) |
| Ezetimibe discontinued | 0.0% (0/122) | 0.6% (2/349) | 0.8% (4/510) | 0.6% (6/981) |
| PCSK9i discontinued | 2.5% (3/122) | 1.1% (4/349) | 0.0% (0/510) | 0.7% (7/981) |

Abbreviations: LDL-C, low-density lipoprotein cholesterol; LLT, lipid-lowering therapy; mAb, monoclonal antibody; PCSK9i, proprotein convertase subtilisin/kexin type 9 inhibitor.

**Supplemental Table 4. Changes in LLT During GOULD EDU by Intervention Step and Original Cohort From the GOULD Parent Study**

|  | **Step 1** | | | | **Step 2** | | | |  |
| --- | --- | --- | --- | --- | --- | --- | --- | --- | --- |
| **'** | **PCSK-9i (N=80 Patients)** | **LDL-C>=100 mg/dL (N=237 Patients)** | **LDL-C 70-99 mg/dL (N=299 Patients)** | **Total (N=616 Patients)** | **PCSK-9i (N=45 Patients)** | **LDL-C>=100 mg/dL (N=118 Patients)** | **LDL-C 70-99 mg/dL (N=219 Patients)** | **Total (N=382 Patients)** | **p-value (comparing Total subjects across Step 1 and Step 2)** |
| No change in LLT | 67.9% (53/78) | 81.5% (190/233) | 86.0% (257/299) | 82.0% (500/610) | 84.1% (37/44) | 87.9% (102/116) | 85.8% (181/211) | 86.3% (320/371) | 0.079 |
| Any change in LLT | 32.1% (25/78) | 18.5% (43/233) | 14.0% (42/299) | 18.0% (110/610) | 15.9% (7/44) | 12.1% (14/116) | 14.2% (30/211) | 13.7% (51/371) | 0.079 |
| Intensification change in LLT |  |  |  |  |  |  |  |  |  |
| Lipid treatment intensification | 12.8% (10/78) | 7.7% (18/233) | 5.0% (15/299) | 7.0% (43/610) | 6.8% (3/44) | 6.0% (7/116) | 7.6% (16/211) | 7.0% (26/371) | 0.981 |
| Lipid treatment de-escalation | 3.8% (3/78) | 6.9% (16/233) | 5.0% (15/299) | 5.6% (34/610) | 0.0% (0/44) | 4.3% (5/116) | 2.8% (6/211) | 3.0% (11/371) | 0.058 |
| No change in treatment intensity | 15.4% (12/78) | 3.9% (9/233) | 4.0% (12/299) | 5.4% (33/610) | 9.1% (4/44) | 1.7% (2/116) | 3.8% (8/211) | 3.8% (14/371) | 0.245 |
| Statin changes | 7.7% (6/78) | 10.7% (25/233) | 9.0% (27/299) | 9.5% (58/610) | 6.8% (3/44) | 4.3% (5/116) | 8.1% (17/211) | 6.7% (25/371) | 0.131 |
| Discontinued | 0.0% (0/78) | 3.4% (8/233) | 2.7% (8/299) | 2.6% (16/610) | 2.3% (1/44) | 2.6% (3/116) | 1.9% (4/211) | 2.2% (8/371) | 0.646 |
| Low intensity | 0.0% (0/78) | 0.0% (0/233) | 0.3% (1/299) | 0.2% (1/610) | 2.3% (1/44) | 0.0% (0/116) | 0.0% (0/211) | 0.3% (1/371) |  |
| Moderate intensity | 0.0% (0/78) | 2.6% (6/233) | 0.7% (2/299) | 1.3% (8/610) | 0.0% (0/44) | 0.0% (0/116) | 0.0% (0/211) | 0.0% (0/371) |  |
| High intensity | 0.0% (0/78) | 0.9% (2/233) | 1.7% (5/299) | 1.1% (7/610) | 0.0% (0/44) | 2.6% (3/116) | 1.9% (4/211) | 1.9% (7/371) |  |
| Down titrated | 0.0% (0/78) | 1.3% (3/233) | 0.7% (2/299) | 0.8% (5/610) | 0.0% (0/44) | 0.9% (1/116) | 1.4% (3/211) | 1.1% (4/371) | 0.680 |
| High to moderate intensity | 0.0% (0/78) | 0.9% (2/233) | 0.7% (2/299) | 0.7% (4/610) | 0.0% (0/44) | 0.0% (0/116) | 0.9% (2/211) | 0.5% (2/371) | 0.820 |
| High to low intensity | 0.0% (0/78) | 0.4% (1/233) | 0.0% (0/299) | 0.2% (1/610) | 0.0% (0/44) | 0.9% (1/116) | 0.0% (0/211) | 0.3% (1/371) | 0.722 |
| Moderate to low intensity | 0.0% (0/78) | 0.0% (0/233) | 0.0% (0/299) | 0.0% (0/610) | 0.0% (0/44) | 0.0% (0/116) | 0.5% (1/211) | 0.3% (1/371) | 0.200 |
| Up titrated | 1.3% (1/78) | 1.7% (4/233) | 2.0% (6/299) | 1.8% (11/610) | 0.0% (0/44) | 0.0% (0/116) | 3.3% (7/211) | 1.9% (7/371) | 0.925 |
| Low to moderate intensity | 0.0% (0/78) | 0.4% (1/233) | 0.0% (0/299) | 0.2% (1/610) | 0.0% (0/44) | 0.0% (0/116) | 0.5% (1/211) | 0.3% (1/371) | 0.722 |
| Low to high intensity | 0.0% (0/78) | 0.0% (0/233) | 0.7% (2/299) | 0.3% (2/610) | 0.0% (0/44) | 0.0% (0/116) | 0.5% (1/211) | 0.3% (1/371) | 0.873 |
| Moderate to high intensity | 1.3% (1/78) | 1.3% (3/233) | 1.3% (4/299) | 1.3% (8/610) | 0.0% (0/44) | 0.0% (0/116) | 2.4% (5/211) | 1.3% (5/371) | 0.962 |
| Changed statin type | 7.7% (6/78) | 9.4% (22/233) | 8.7% (26/299) | 8.9% (54/610) | 6.8% (3/44) | 4.3% (5/116) | 5.7% (12/211) | 5.4% (20/371) | 0.046 |
| Added | 6.4% (5/78) | 2.6% (6/233) | 1.3% (4/299) | 2.5% (15/610) | 4.5% (2/44) | 0.0% (0/116) | 0.9% (2/211) | 1.1% (4/371) | 0.128 |
| Low intensity | 1.3% (1/78) | 0.4% (1/233) | 0.0% (0/299) | 0.3% (2/610) | 0.0% (0/44) | 0.0% (0/116) | 0.0% (0/211) | 0.0% (0/371) |  |
| Moderate intensity | 3.8% (3/78) | 1.3% (3/233) | 0.3% (1/299) | 1.1% (7/610) | 2.3% (1/44) | 0.0% (0/116) | 0.5% (1/211) | 0.5% (2/371) |  |
| High intensity | 1.3% (1/78) | 0.9% (2/233) | 1.0% (3/299) | 1.0% (6/610) | 2.3% (1/44) | 0.0% (0/116) | 0.5% (1/211) | 0.5% (2/371) |  |
| Ezetimibe changes | 3.8% (3/78) | 2.6% (6/233) | 2.7% (8/299) | 2.8% (17/610) | 0.0% (0/44) | 2.6% (3/116) | 3.3% (7/211) | 2.7% (10/371) | 0.932 |
| Discontinued | 0.0% (0/78) | 0.9% (2/233) | 1.3% (4/299) | 1.0% (6/610) | 0.0% (0/44) | 0.0% (0/116) | 0.0% (0/211) | 0.0% (0/371) | 0.055 |
| Added | 3.8% (3/78) | 1.7% (4/233) | 1.3% (4/299) | 1.8% (11/610) | 0.0% (0/44) | 2.6% (3/116) | 3.3% (7/211) | 2.7% (10/371) | 0.349 |
| PCSK9i changes | 16.7% (13/78) | 3.4% (8/233) | 0.3% (1/299) | 3.6% (22/610) | 9.1% (4/44) | 4.3% (5/116) | 0.5% (1/211) | 2.7% (10/371) | 0.436 |
| Discontinued | 3.8% (3/78) | 1.3% (3/233) | 0.0% (0/299) | 1.0% (6/610) | 0.0% (0/44) | 0.9% (1/116) | 0.0% (0/211) | 0.3% (1/371) | 0.198 |
| Added | 1.3% (1/78) | 0.9% (2/233) | 0.3% (1/299) | 0.7% (4/610) | 2.3% (1/44) | 2.6% (3/116) | 0.5% (1/211) | 1.3% (5/371) | 0.270 |
| Up titrated (only for Alirocumab) | 0.0% (0/78) | 0.0% (0/233) | 0.0% (0/299) | 0.0% (0/610) | 0.0% (0/44) | 0.9% (1/116) | 0.0% (0/211) | 0.3% (1/371) | 0.200 |
| Down titrated (only for Alirocumab) | 0.0% (0/78) | 0.0% (0/233) | 0.0% (0/299) | 0.0% (0/610) | 0.0% (0/44) | 0.0% (0/116) | 0.0% (0/211) | 0.0% (0/371) | -- |
| Other change (eg. the PCSK9i dosage/frequency is unclassified) | 11.5% (9/78) | 1.3% (3/233) | 0.0% (0/299) | 2.0% (12/610) | 6.8% (3/44) | 0.0% (0/116) | 0.0% (0/211) | 0.8% (3/371) | 0.152 |
| Fish oil changes | 6.4% (5/78) | 1.7% (4/233) | 3.0% (9/299) | 3.0% (18/610) | 2.3% (1/44) | 0.0% (0/116) | 3.3% (7/211) | 2.2% (8/371) | 0.453 |
| Discontinued | 5.1% (4/78) | 0.9% (2/233) | 1.7% (5/299) | 1.8% (11/610) | 0.0% (0/44) | 0.0% (0/116) | 2.8% (6/211) | 1.6% (6/371) | 0.829 |
| Added | 1.3% (1/78) | 0.9% (2/233) | 1.3% (4/299) | 1.1% (7/610) | 2.3% (1/44) | 0.0% (0/116) | 0.5% (1/211) | 0.5% (2/371) | 0.332 |
| Other lipid lowering medications change^1^ | 3.8% (3/78) | 2.1% (5/233) | 2.0% (6/299) | 2.3% (14/610) | 0.0% (0/44) | 1.7% (2/116) | 0.5% (1/211) | 0.8% (3/371) | 0.084 |
| Discontinued an other lipid lowering medication | 0.0% (0/78) | 1.3% (3/233) | 1.3% (4/299) | 1.1% (7/610) | 0.0% (0/44) | 0.9% (1/116) | 0.0% (0/211) | 0.3% (1/371) | 0.138 |
| Added an other lipid lowering medication | 3.8% (3/78) | 0.9% (2/233) | 0.7% (2/299) | 1.1% (7/610) | 0.0% (0/44) | 0.9% (1/116) | 0.5% (1/211) | 0.5% (2/371) | 0.332 |

Note: The end of study data are from the last GOULD EDU chart review.

For subjects with multiple statin records at the end of study, only the record with the highest intensity is presented in the table.

1Other LLTs included: fibrate, niacin, mipomersen, lomitapide, cholestyramine, colesevelam, colestipol, and other unclassified LLTs except statins, ezetimibe, PCSK9i, and fish oil.

Statin intensity per day was based on 2013 ACC AHA Guideline on Treatment of Blood Cholesterol to Reduce ASCVD Risk in Adults, as the following: High-intensity: atorvastatin 40 mg-80 mg; rosuvastatin 20 mg- 40 mg; simvastatin 80 mg; Moderate-intensity: atorvastatin 10 mg-20 mg; rosuvastatin 5 mg-10 mg; simvastatin 20 mg-40 mg; pravastatin 40 mg-80 mg; lovastatin 40 mg; fluvastatin 40 mg-80 mg; pitavastatin 2 mg-4 mg; Low-intensity: simvastatin 10 mg, pravastatin 10 mg-20 mg; lovastatin 20 mg; fluvastatin 20 mg-40 mg; pitavastatin 1 mg.

Undefined statin doses were classified as follows: Higher than doses in high-intensity = high-intensity; Between high-intensity and moderate-intensity = moderate-intensity; Between moderate-intensity and low-intensity = low-intensity; Lower than low-intensity = low-intensity.

Abbreviations: ACC, American College of Cardiology; AHA, American Heart Association; ASCVD, atherosclerotic cardiovascular disease; LDL-C, low-density lipoprotein cholesterol; LLT, lipid-lowering therapy; PCSK9i, proprotein convertase subtilisin/kexin type 9 inhibitor.

**Supplemental Table 5. Physician Questionnaire Responses**

1. **Clinical guidelines**

| **Question** | **Before educational intervention (N=49)** | **After educational intervention (N=49)** |
| --- | --- | --- |
| **Clinical guidelines, % (n/N) choosing each response** |  |  |
| How familiar are you with the 2018 ACC/AHA cholesterol guidelines? |  |  |
| Not familiar | 0.0% (0/49) | 0.0% (0/49) |
| Somewhat familiar | 24.5% (12/49) | 22.4% (11/49) |
| Very familiar | 75.5% (37/49) | 77.6% (38/49) |
| How familiar are you with the 2019 ESC cholesterol guidelines? |  |  |
| Not familiar | 14.3% (7/49) | 8.2% (4/49) |
| Somewhat familiar | 38.8% (19/49) | 40.8% (20/49) |
| Very familiar | 46.9% (23/49) | 51.0% (25/49) |
| How often do you apply the 2018 ACC/AHA Cholesterol Guidelines in your day-to-day clinical practice? |  |  |
| Never | 2.0% (1/49) | 0.0% (0/49) |
| Rarely | 0.0% (0/49) | 0.0% (0/49) |
| Sometimes | 16.3% (8/49) | 8.2% (4/49) |
| Often | 46.9% (23/49) | 65.3% (32/49) |
| All of the time | 34.7% (17/49) | 26.5% (13/49) |
| How often do you apply the 2019 ESC Cholesterol Guidelines in your day-to-day clinical practice? |  |  |
| Never | 18.4% (9/49) | 8.2% (4/49) |
| Rarely | 4.1% (2/49) | 6.1% (3/49) |
| Sometimes | 24.5% (12/49) | 38.8% (19/49) |
| Often | 34.7% (17/49) | 28.6% (14/49) |
| All of the time | 18.4% (9/49) | 18.4% (9/49) |

1. **LDL-C goals and low LDL-C**

| **Question** | **Before educational intervention (N=49)** | **After educational intervention (N=49)** |
| --- | --- | --- |
| **What is the LDL-C goal that you aim to achieve with cholesterol-lowering therapy for your patients with ASCVD?, % (n/N) choosing each response** |  |  |
| LDL-C goal to achieve with cholesterol-lowering therapy |  |  |
| Less than 50 mg/dL | 12.2% (6/49) | 18.4% (9/49) |
| Less than 70 mg/dL | 69.4% (34/49) | 65.3% (32/49) |
| Less than 100 mg/dL | 8.2% (4/49) | 4.1% (2/49) |
| Less than 130 mg/dL | 0.0% (0/49) | 0.0% (0/49) |
| It depends on patient’s other risk factors (e.g., if they have diabetes) | 10.2% (5/49) | 8.2% (4/49) |
| Do not use LDL-C goals for patients with ASCVD | 0.0% (0/49) | 4.1% (2/49) |
| What do you usually do when a patient has very low LDL cholesterol—for example, less than 25 mg/dL? |  |  |
| Make no change to lipid-lowering therapy | 55.1% (27/49) | 63.3% (31/49) |
| Reduce the lipid-lowering medication intensity | 38.8% (19/49) | 20.4% (10/49) |
| Discontinue one or more lipid-lowering medication | 6.1% (3/49) | 10.2% (5/49) |
| Switch lipid-lowering drugs | 0.0% (0/49) | 2.0% (1/49) |
| Other | 0.0% (0/49) | 4.1% (2/49) |

1. **Lipid measurement during statin treatment**

| **Question** | **Before educational intervention (N=49)** | **After educational intervention (N=49)** |
| --- | --- | --- |
| **Lipid measurement during statin treatment** |  |  |
| After a patient with ASCVD first initiates statins, how long do you wait until you first reassess their lipid levels?, % (n/N) choosing each response |  |  |
| Less than three months | 42.9% (21/49) | 51.0% (25/49) |
| Three to six months | 53.1% (26/49) | 40.8% (20/49) |
| Six months to one year | 2.0% (1/49) | 8.2% (4/49) |
| More than one year | 0.0% (0/49) | 0.0% (0/49) |
| Never | 2.0% (1/49) | 0.0% (0/49) |
| Please rate the importance of reasons for measuring lipid levels after a patient with ASCVD initiates a statin, (0 [not important at all] to 10 [extremely important] scale), Mean ± SD (N) |  |  |
| How important is measuring lipids for assessing medication adherence? | 9.3±0.9 (49) | 8.9±1.6 (49) |
| How important is measuring lipids for assessing patient response to therapy (e.g., a 30% or larger decline from pre-treatment levels)? | 9.2±1.0 (49) | 9.1±1.3 (49) |
| How important is measuring lipids for determining whether an LDL-C target (e.g., < 70 mg/dL) is achieved? | 9.3±1.0 (49) | 9.1±1.0 (49) |
| How important is measuring lipids for calculating predicted ASCVD risk? | 7.8±2.3 (48) | 7.6±2.2 (48) |
| For patients with ASCVD who have been taking statins for more than one year, how often do you measure their lipid levels?, % (n/N) choosing each response |  |  |
| At least every six months | 42.9% (21/49) | 34.7% (17/49) |
| Every six months to one year | 46.9% (23/49) | 61.2% (30/49) |
| Less frequently than once per year | 8.2% (4/49) | 4.1% (2/49) |
| Never | 2.0% (1/49) | 0.0% (0/49) |
| For what percentage of your patients with ASCVD taking statins do you measure the following parameters? |  |  |
| Total Cholesterol |  |  |
| 0% | 0.0% (0/49) | 0.0% (0/49) |
| 1% to 24% | 0.0% (0/49) | 0.0% (0/49) |
| 25% to 49% | 0.0% (0/49) | 2.0% (1/49) |
| 50% to 74% | 4.1% (2/49) | 4.1% (2/49) |
| 75% to 99% | 20.4% (10/49) | 18.4% (9/49) |
| 100% | 75.5% (37/49) | 75.5% (37/49) |
| HDL-Cholesterol |  |  |
| 0% | 0.0% (0/49) | 0.0% (0/49) |
| 1% to 24% | 0.0% (0/49) | 0.0% (0/49) |
| 25% to 49% | 0.0% (0/49) | 2.0% (1/49) |
| 50% to 74% | 4.1% (2/49) | 4.1% (2/49) |
| 75% to 99% | 24.5% (12/49) | 20.4% (10/49) |
| 100% | 71.4% (35/49) | 73.5% (36/49) |
| Triglycerides |  |  |
| 0% | 0.0% (0/49) | 0.0% (0/49) |
| 1% to 24% | 0.0% (0/49) | 0.0% (0/49) |
| 25% to 49% | 2.0% (1/49) | 0.0% (0/49) |
| 50% to 74% | 4.1% (2/49) | 10.2% (5/49) |
| 75% to 99% | 22.4% (11/49) | 18.4% (9/49) |
| 100% | 69.4% (34/49) | 71.4% (35/49) |
| Lp (a) |  |  |
| 0% | 16.3% (8/49) | 12.2% (6/49) |
| 1% to 24% | 38.8% (19/49) | 36.7% (18/49) |
| 25% to 49% | 12.2% (6/49) | 16.3% (8/49) |
| 50% to 74% | 12.2% (6/49) | 20.4% (10/49) |
| 75% to 99% | 8.2% (4/49) | 4.1% (2/49) |
| 100% | 12.2% (6/49) | 10.2% (5/49) |
| ApoB |  |  |
| 0% | 22.4% (11/49) | 26.5% (13/49) |
| 1% to 24% | 38.8% (19/49) | 30.6% (15/49) |
| 25% to 49% | 10.2% (5/49) | 18.4% (9/49) |
| 50% to 74% | 14.3% (7/49) | 18.4% (9/49) |
| 75% to 99% | 6.1% (3/49) | 2.0% (1/49) |
| 100% | 6.1% (3/49) | 4.1% (2/49) |
| LDL particle number |  |  |
| 0% | 14.3% (7/49) | 20.4% (10/49) |
| 1% to 24% | 34.7% (17/49) | 32.7% (16/49) |
| 25% to 49% | 4.1% (2/49) | 6.1% (3/49) |
| 50% to 74% | 14.3% (7/49) | 16.3% (8/49) |
| 75% to 99% | 12.2% (6/49) | 8.2% (4/49) |
| 100% | 20.4% (10/49) | 16.3% (8/49) |
| C-reactive protein |  |  |
| 0% | 12.2% (6/49) | 18.4% (9/49) |
| 1% to 24% | 32.7% (16/49) | 32.7% (16/49) |
| 25% to 49% | 20.4% (10/49) | 6.1% (3/49) |
| 50% to 74% | 18.4% (9/49) | 20.4% (10/49) |
| 75% to 99% | 8.2% (4/49) | 16.3% (8/49) |
| 100% | 8.2% (4/49) | 6.1% (3/49) |
| Do you use any other biomarkers, such as coronary calcium or high sensitivity c-reactive protein to guide the intensity of lipid-lowering therapy for your patients with ASCVD? | 59.2% (29/49) | 67.3% (33/49) |

1. **Treating ASCVD patients with maximally tolerated statin therapy who have high LDL-C measurement**

| **Question** | **Before educational intervention (N=49)** | **After educational intervention (N=49)** |
| --- | --- | --- |
| **The following questions are related to patients with ASCVD taking a maximally tolerated statin dose having high LDL-C measurement, % (n/N) choosing each response** |  |  |
| How often do you wait and re-measure lipids at a follow-up visit? |  |  |
| Never | 2.0% (1/49) | 2.0% (1/49) |
| Rarely | 4.1% (2/49) | 2.0% (1/49) |
| Some of the time | 24.5% (12/49) | 26.5% (13/49) |
| Most of the time | 36.7% (18/49) | 32.7% (16/49) |
| Always | 32.7% (16/49) | 36.7% (18/49) |
| How often do you discuss adherence issues? |  |  |
| Never | 0.0% (0/49) | 0.0% (0/49) |
| Rarely | 0.0% (0/49) | 0.0% (0/49) |
| Some of the time | 12.2% (6/49) | 8.2% (4/49) |
| Most of the time | 32.7% (16/49) | 46.9% (23/49) |
| Always | 55.1% (27/49) | 44.9% (22/49) |
| How frequently do you consider adding a non-statin lipid lowering drug? |  |  |
| Never | 0.0% (0/49) | 0.0% (0/49) |
| Rarely | 0.0% (0/49) | 6.1% (3/49) |
| Some of the time | 53.1% (26/49) | 53.1% (26/49) |
| Most of the time | 30.6% (15/49) | 18.4% (9/49) |
| Always | 16.3% (8/49) | 22.4% (11/49) |
| How often do you discuss lifestyle modification? |  |  |
| Never | 0.0% (0/49) | 0.0% (0/49) |
| Rarely | 2.0% (1/49) | 0.0% (0/49) |
| Some of the time | 4.1% (2/49) | 4.1% (2/49) |
| Most of the time | 22.4% (11/49) | 26.5% (13/49) |
| Always | 71.4% (35/49) | 69.4% (34/49) |
| How often do you change statin type? |  |  |
| Never | 0.0% (0/49) | 0.0% (0/49) |
| Rarely | 2.0% (1/49) | 10.2% (5/49) |
| Some of the time | 85.7% (42/49) | 79.6% (39/49) |
| Most of the time | 10.2% (5/49) | 8.2% (4/49) |
| Always | 2.0% (1/49) | 2.0% (1/49) |
| In your patients who are non-adherent to their statins, what is the most common reason for non-adherence? |  |  |
| They don't believe the medication works | 2.0% (1/49) | 0.0% (0/49) |
| They don't have access to a pharmacy | 0.0% (0/49) | 0.0% (0/49) |
| They can't afford the medication | 10.2% (5/49) | 4.1% (2/49) |
| They experienced side effects | 79.6% (39/49) | 81.6% (40/49) |
| They don't like taking medications | 4.1% (2/49) | 14.3% (7/49) |
| Other | 4.1% (2/49) | 0.0% (0/49) |

1. **Statin therapy evidence and side effects**

| **Question** | **Before educational intervention (N=49)** | **After educational intervention (N=49)** |
| --- | --- | --- |
| **Statin therapy evidence and side effects** |  |  |
| In your opinion, how strong is the scientific evidence supporting the use of high intensity statins vs. low/moderate intensity statins among patients with ASCVD?, % (n/N) choosing each response |  |  |
| Very strong | 44.9% (22/49) | 44.9% (22/49) |
| Strong | 38.8% (19/49) | 36.7% (18/49) |
| Moderate | 16.3% (8/49) | 12.2% (6/49) |
| Weak | 0.0% (0/49) | 4.1% (2/49) |
| Very Weak | 0.0% (0/49) | 0.0% (0/49) |
| Refused | 0.0% (0/49) | 2.0% (1/49) |
| In your opinion, how strong is the scientific evidence supporting the use of high intensity statins for cardiovascular disease risk reduction among patients 75 years and older with ASCVD?, % (n/N) choosing each response |  |  |
| Very strong | 14.3% (7/49) | 12.2% (6/49) |
| Strong | 49.0% (24/49) | 49.0% (24/49) |
| Moderate | 24.5% (12/49) | 30.6% (15/49) |
| Weak | 12.2% (6/49) | 8.2% (4/49) |
| Very Weak | 0.0% (0/49) | 0.0% (0/49) |
| In general, what percentage of patients taking statins experience side effects?, scale of 1-100%, mean ± SD (N) | 18.9±15.3 (47) | 18.0±14.1 (49) |
| In general, are you concerned about prescribing high intensity statins due to side effects occurring?, % (n/N) responding yes | 26.5% (13/49) | 28.6% (14/49) |
| Among patients who have muscle pain or aches while taking statins, what percentage do you think is due to the statin?, scale of 1-100% percent, mean ± SD (N) | 37.9±27.0 (47) | 28.0±19.9 (48) |
| Among patients who have muscle pain or aches within three months after initiating a statin, what percentage do you think is due to the statin?, scale of 1-100%, mean ± SD (N) | 40.6±28.1 (48) | 33.8±21.8 (49) |
| How often do you take each of the following actions when you have a patient with ASCVD who reports side effects while taking statins?, % (n/N) choosing each response |  |  |
| Down-titrate intensity |  |  |
| Never | 2.0% (1/49) | 2.0% (1/49) |
| Rarely | 36.7% (18/49) | 44.9% (22/49) |
| Some of the time | 12.2% (6/49) | 14.3% (7/49) |
| Most of the time | 42.9% (21/49) | 32.7% (16/49) |
| Always | 6.1% (3/49) | 4.1% (2/49) |
| Missing | 0.0% (0/49) | 2.0% (1/49) |
| Discontinue treatment |  |  |
| Never | 4.1% (2/49) | 6.1% (3/49) |
| Rarely | 53.1% (26/49) | 38.8% (19/49) |
| Some of the time | 38.8% (19/49) | 51.0% (25/49) |
| Most of the time | 4.1% (2/49) | 2.0% (1/49) |
| Always | 0.0% (0/49) | 2.0% (1/49) |
| Educate patients on the importance of statins |  |  |
| Never | 0.0% (0/49) | 0.0% (0/49) |
| Rarely | 12.2% (6/49) | 10.2% (5/49) |
| Some of the time | 0.0% (0/49) | 0.0% (0/49) |
| Most of the time | 12.2% (6/49) | 10.2% (5/49) |
| Always | 75.5% (37/49) | 79.6% (39/49) |
| Switch statin type |  |  |
| Never | 0.0% (0/49) | 0.0% (0/49) |
| Rarely | 57.1% (28/49) | 57.1% (28/49) |
| Some of the time | 0.0% (0/49) | 8.2% (4/49) |
| Most of the time | 40.8% (20/49) | 30.6% (15/49) |
| Always | 2.0% (1/49) | 4.1% (2/49) |
| Add a supplement (e.g., CO-Q10 or vitamin D) |  |  |
| Never | 8.2% (4/49) | 8.2% (4/49) |
| Rarely | 38.8% (19/49) | 40.8% (20/49) |
| Some of the time | 18.4% (9/49) | 18.4% (9/49) |
| Most of the time | 30.6% (15/49) | 26.5% (13/49) |
| Always | 4.1% (2/49) | 6.1% (3/49) |
| How often do you take each of the following actions when re-challenging a patient with ASCVD on a statin, % (n/N) choosing each response |  |  |
| Restart therapy at a lower dose |  |  |
| Never | 2.0% (1/49) | 0.0% (0/49) |
| Rarely | 44.9% (22/49) | 44.9% (22/49) |
| Some of the time | 6.1% (3/49) | 6.1% (3/49) |
| Most of the time | 38.8% (19/49) | 42.9% (21/49) |
| Always | 8.2% (4/49) | 6.1% (3/49) |
| Counsel the patient on adherence |  |  |
| Never | 0.0% (0/49) | 0.0% (0/49) |
| Rarely | 18.4% (9/49) | 14.3% (7/49) |
| Some of the time | 4.1% (2/49) | 2.0% (1/49) |
| Most of the time | 16.3% (8/49) | 20.4% (10/49) |
| Always | 61.2% (30/49) | 63.3% (31/49) |
| Use a different statin type |  |  |
| Never | 0.0% (0/49) | 0.0% (0/49) |
| Rarely | 38.8% (19/49) | 49.0% (24/49) |
| Some of the time | 4.1% (2/49) | 2.0% (1/49) |
| Most of the time | 46.9% (23/49) | 40.8% (20/49) |
| Always | 10.2% (5/49) | 8.2% (4/49) |
| Use non-statin LLT along with the statin |  |  |
| Never | 0.0% (0/49) | 0.0% (0/49) |
| Rarely | 55.1% (27/49) | 65.3% (32/49) |
| Some of the time | 10.2% (5/49) | 8.2% (4/49) |
| Most of the time | 30.6% (15/49) | 20.4% (10/49) |
| Always | 4.1% (2/49) | 6.1% (3/49) |
| Advise the patient to take their medication every other day |  |  |
| Never | 12.2% (6/49) | 12.2% (6/49) |
| Rarely | 51.0% (25/49) | 49.0% (24/49) |
| Some of the time | 22.4% (11/49) | 24.5% (12/49) |
| Most of the time | 12.2% (6/49) | 14.3% (7/49) |
| Always | 2.0% (1/49) | 0.0% (0/49) |
| Advise the patient to take a supplement with their statin (e.g., add CO-Q10) |  |  |
| Never | 8.2% (4/49) | 6.1% (3/49) |
| Rarely | 46.9% (23/49) | 32.7% (16/49) |
| Some of the time | 20.4% (10/49) | 26.5% (13/49) |
| Most of the time | 18.4% (9/49) | 24.5% (12/49) |
| Always | 6.1% (3/49) | 10.2% (5/49) |

1. **Frequency of prescribing non-statin LLT**

| **Question** | **Before educational intervention (N=49)** | **After educational intervention (N=49)** |
| --- | --- | --- |
| **The following question is about your use of non-statin lipid-lowering therapies, including ezetimibe, niacin, bile acid sequestrants, fibrates, and omega-3 fatty acids.  We will ask separately about PCSK9 inhibitors in the next set of questions.** |  |  |
| **When you encounter each of the following scenarios, how often do you prescribe non-statin lipid-lowering therapy, % (n/N) choosing each response** |  |  |
| Patients cannot tolerate statins |  |  |
| Never | 0.0% (0/49) | 0.0% (0/49) |
| Rarely | 24.5% (12/49) | 24.5% (12/49) |
| Some of the time | 4.1% (2/49) | 2.0% (1/49) |
| Most of the time | 36.7% (18/49) | 40.8% (20/49) |
| Always | 34.7% (17/49) | 32.7% (16/49) |
| LDL-C remains high despite statin use |  |  |
| Never | 0.0% (0/49) | 0.0% (0/49) |
| Rarely | 18.4% (9/49) | 30.6% (15/49) |
| Some of the time | 4.1% (2/49) | 0.0% (0/49) |
| Most of the time | 51.0% (25/49) | 36.7% (18/49) |
| Always | 26.5% (13/49) | 32.7% (16/49) |
| Further cardiovascular disease risk reduction above and beyond statin use |  |  |
| Never | 0.0% (0/49) | 0.0% (0/49) |
| Rarely | 34.7% (17/49) | 38.8% (19/49) |
| Some of the time | 2.0% (1/49) | 6.1% (3/49) |
| Most of the time | 49.0% (24/49) | 38.8% (19/49) |
| Always | 14.3% (7/49) | 16.3% (8/49) |

1. **PCSK9 inhibitor monoclonal antibody treatments**

| **Question** | **Before educational intervention (N=49)** | **After educational intervention (N=49)** |
| --- | --- | --- |
| **The following questions are about a specific non-statin lipid-lowering therapy—PCSK9 inhibitors, (0–10 scale), Mean ± SD (N)** |  |  |
| How familiar are you with PCSK9 inhibitors? (0 [not familiar at all] to 10 [extremely familiar] scale) | 9.1±1.3 (49) | 8.9±1.5 (49) |
| Have you considered prescribing a PCSK9 inhibitor for any of your patients? % (n/N) responding yes | 93.9% (46/49) | 98.0% (48/49) |
| Have you prescribed a PCSK9 inhibitor for any of your patients? % (n/N) responding yes | 95.9% (47/49) | 89.8% (44/49) |
| Considering patients for whom you considered a PCSK9 inhibitor, but did not prescribe one, please rate the importance of each of the following in your decision not to prescribe a PCSK9 inhibitor for some of your patients with ASCVD. (0 [not important at all] to 10 [extremely important] scale) |  |  |
| How important were challenges obtaining insurance company approval in your decision not to prescribe a PCSK9i? | 7.6±2.7 (49) | 7.2±2.9 (49) |
| How important was cost in your decision not to prescribe a PCSK9i? | 7.1±2.9 (49) | 6.8±2.9 (49) |
| How important were concerns that LDL cholesterol would become too low in your decision not to prescribe a PCSK9 inhibitor for some of your patients? | 2.4±2.4 (49) | 2.3±2.0 (49) |
| How important were questions about the efficacy of PCSK9i for ASCVD reduction in your decision not to prescribe a PCSK9i? | 2.8±2.7 (49) | 2.9±2.6 (49) |
| How important were concerns about side effects in your decision not to prescribe a PCSK9i for some of your patients with ASCVD? | 2.7±2.4 (49) | 3.0±2.5 (49) |
| How important was the injectable delivery method in your decision not to prescribe a PCSK9i inhibitor for some of your patients with ASCVD? | 3.1±2.6 (49) | 3.5±2.5 (49) |
| How important was patient preference in your decision not to prescribe a PCSK9i for some of your patients with ASCVD? | 5.5±2.9 (49) | 5.6±2.7 (49) |
| How important was your lack of familiarity with PCSK9i in your decision not to prescribe a PCSK9i for some of your patients with ASCVD? | 2.2±2.4 (49) | 2.8±2.6 (49) |

1. **Familial hypercholesterolemia**

| **Question** | **Before educational intervention (N=49)** | **After educational intervention (N=49)** |
| --- | --- | --- |
| Some patients with very high LDL-cholesterol have familial hypercholesterolemia. In general, what percentage of patients with an LDL-C above 190 mg/dL do you think have familial hypercholesterolemia? (0–10 scale), Mean ± SD (N) | 49.6±31.0 (49) | 51.3±29.3 (49) |

1. **Patient education**

| **Question** | **Before educational intervention (N=49)** | **After educational intervention (N=49)** |
| --- | --- | --- |
| **Patient education, % (n/N) choosing each response** |  |  |
| Approximately how long do you spend on a regular follow-up visit with a patient with ASCVD? |  |  |
| Less than 5 minutes | 0.0% (0/49) | 0.0% (0/49) |
| 5 to less than 10 minutes | 6.1% (3/49) | 4.1% (2/49) |
| 10 to less than 15 minutes | 14.3% (7/49) | 20.4% (10/49) |
| 15 to less than 20 minutes | 49.0% (24/49) | 30.6% (15/49) |
| 20 minutes or more | 30.6% (15/49) | 44.9% (22/49) |
| Do you spend time providing your patients education on lifestyle modification for lowering LDL-C? (yes) | 95.9% (47/49) | 95.9% (47/49) |
| Do you spend time on education about side effects of lipid-lowering therapy? (yes) | 91.8% (45/49) | 95.9% (47/49) |

1. **Treatment targets**

| **Question** | **Before educational intervention (N=49)** | **After educational intervention (N=49)** |
| --- | --- | --- |
| **After your LDL-C goals are achieved, what is the next biomarker in a patient’s profile that you aim to target with therapy?** |  |  |
| Non-HDL-cholesterol | 20.4% (10/49) | 18.4% (9/49) |
| ApoB | 8.2% (4/49) | 8.2% (4/49) |
| Lp(a) | 8.2% (4/49) | 6.1% (3/49) |
| Total cholesterol | 6.1% (3/49) | 6.1% (3/49) |
| Triglycerides | 42.9% (21/49) | 44.9% (22/49) |
| None—only focus on LDL-C | 14.3% (7/49) | 16.3% (8/49) |
| What is your perspective on optimizing triglyceride levels in your patients? |  |  |
| I do not treat triglycerides | 2.0% (1/49) | 0.0% (0/49) |
| I only target triglycerides if they are very high (i.e. >500 mg/dL) | 12.2% (6/49) | 12.2% (6/49) |
| I treat moderate hypertriglyceridemia (150-499 mg/dL) in very high risk patients | 36.7% (18/49) | 38.8% (19/49) |
| I treat moderate hypertriglyceridemia (150-499 mg/dL) in moderate risk patients | 49.0% (24/49) | 49.0% (24/49) |
| Which is your preferred medication to target triglycerides? |  |  |
| Niacin | 2.0% (1/49) | 4.1% (2/49) |
| Fibrates | 24.5% (12/49) | 28.6% (14/49) |
| Vascepa | 53.1% (26/49) | 51.0% (25/49) |
| Lovaza | 14.3% (7/49) | 10.2% (5/49) |
| Other EPA/DHA high dose prescription therapy | 0.0% (0/49) | 0.0% (0/49) |
| None of above | 6.1% (3/49) | 6.1% (3/49) |
| What is your approach to measuring Lp(a)? |  |  |
| I never measure it | 20.4% (10/49) | 16.3% (8/49) |
| I measure it rarely in high risk ASCVD patients | 36.7% (18/49) | 49.0% (24/49) |
| I measure it regularly in high risk ASCVD patients | 28.6% (14/49) | 22.4% (11/49) |
| I measure it regularly in all my ASCVD patients | 14.3% (7/49) | 12.2% (6/49) |

1. **Effect of GOULD results on physician practice**

| **Question** | **Before educational intervention (N=49)** | **After educational intervention (N=49)** |
| --- | --- | --- |
| **Effect of GOULD results on my practice, % (n/N) choosing each response** |  |  |
| Was the rate of lipid lowering therapy intensification you saw in GOULD what you expected? % (n/N) |  |  |
| Yes | 91.8% (45/49) | 75.5% (37/49) |
| No | 8.2% (4/49) | 24.5% (12/49) |
| Would you make any changes in your practice based on the data you see? |  |  |
| No changes to treatment | 30.6% (15/49) | 40.8% (20/49) |
| Treat LDL-C more aggressively and aim to get ASCVD patients to LDL < 70 | 40.8% (20/49) | 26.5% (13/49) |
| Aim to treat very high risk ASCVD patients to an LDL-C threshold < 55 mg/dL | 26.5% (13/49) | 28.6% (14/49) |
| Not target LDL levels, but aim to increase use of intensive statin therapy | 2.0% (1/49) | 4.1% (2/49) |

Abbreviations: ACC, American College of Cardiology; AHA, American Heart Association; ApoB, apolipoprotein B; ASCVD, atherosclerotic cardiovascular disease; CO-Q10, coenzyme Q10; DHA, docosahexaenoic acid; EPA, eicosapentaenoic acid; ESC, European Society of Cardiology; HDL, high density lipoprotein; LDL-C, low density lipoprotein-cholesterol; LLT, lipid lowering therapy; Lp(a), lipoprotein(a); PCSK9i, proprotein convertase subtilisin/kexin type 9 inhibitor; SD, standard deviation.

**Supplemental Figure 1. Study Design of GOULD and GOULD EDU**


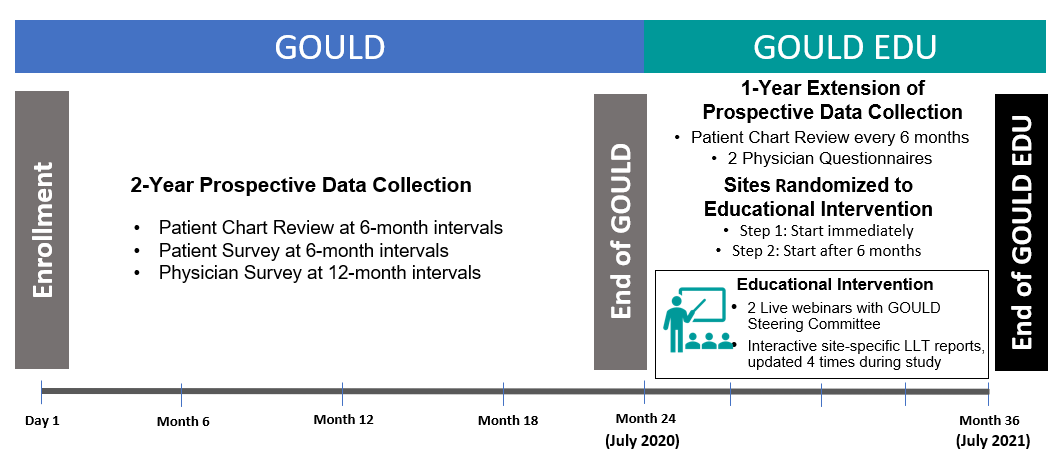


Abbreviations: LLT, lipid-lowering therapy.

**Supplemental Figure 2. Sample Figures From Interactive GOULD Registry Site Report**

***
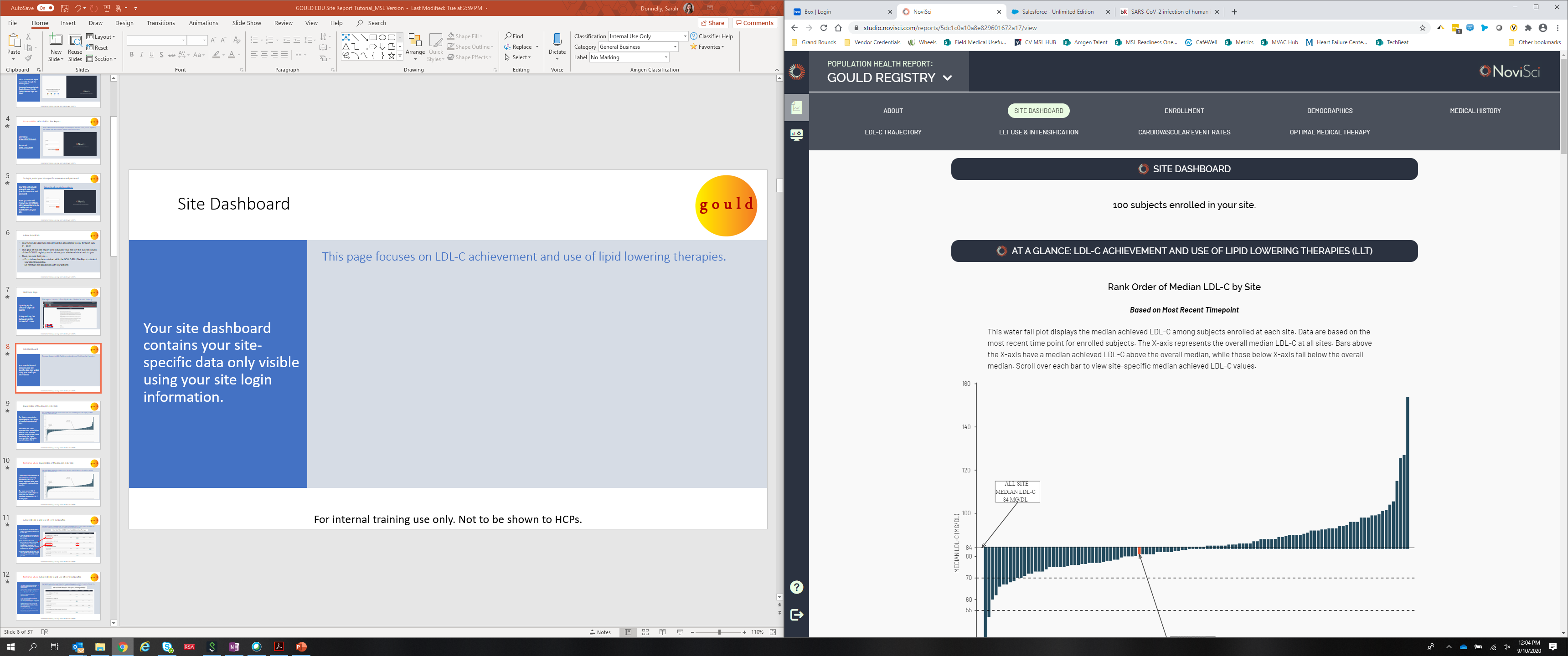
***

***
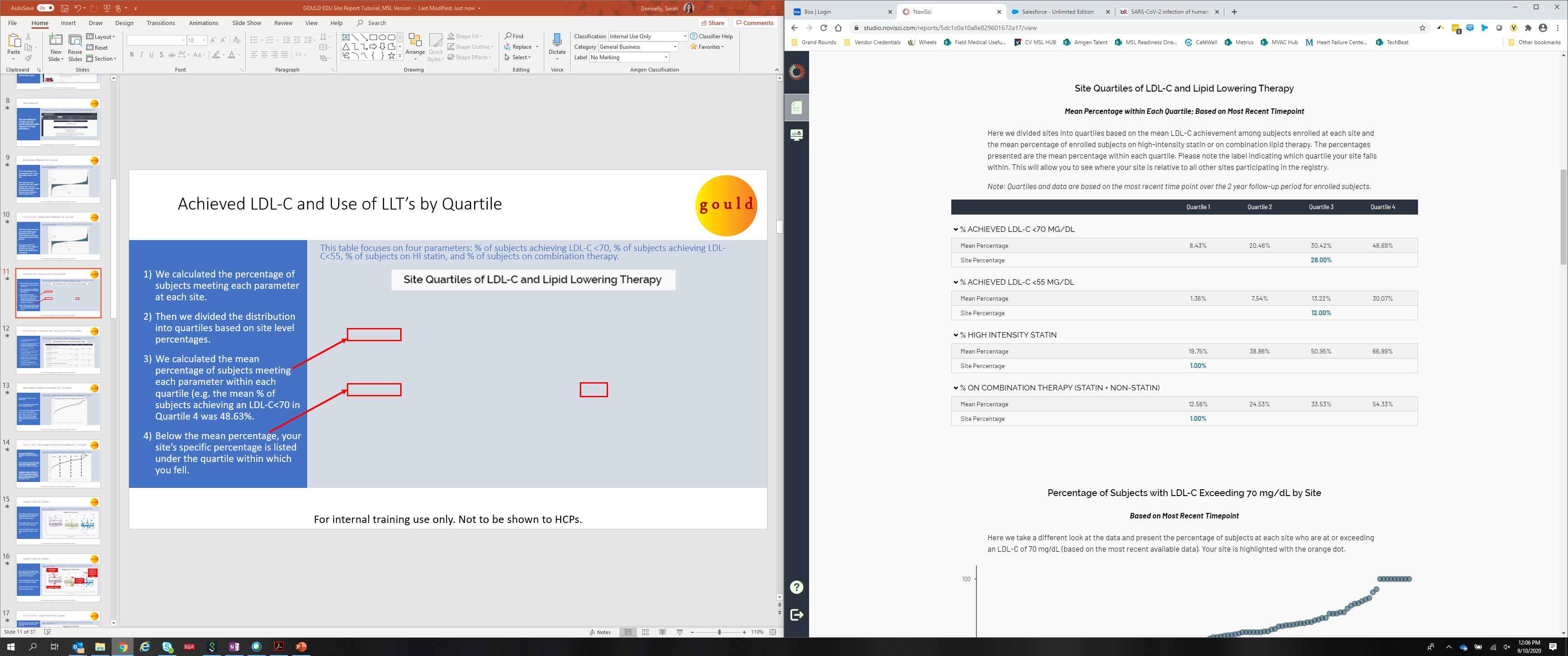
***

***
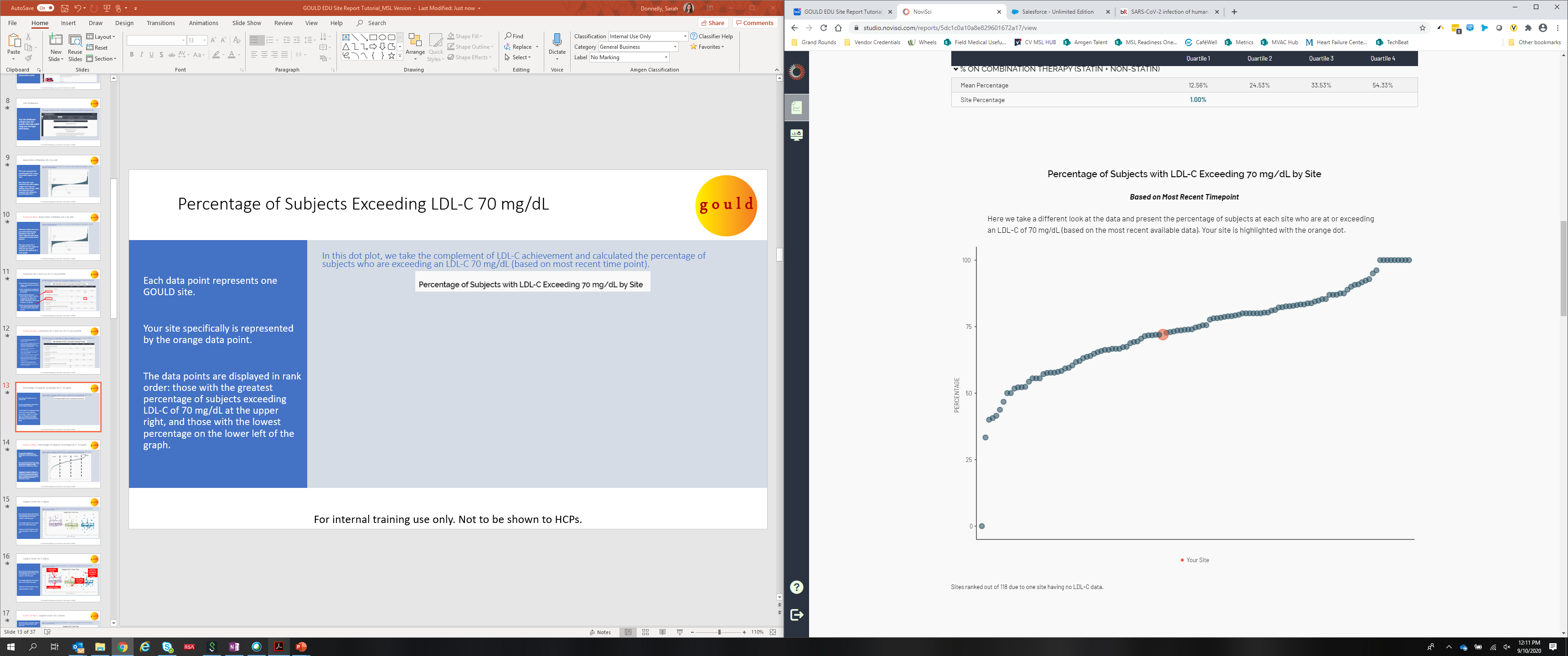
***

***
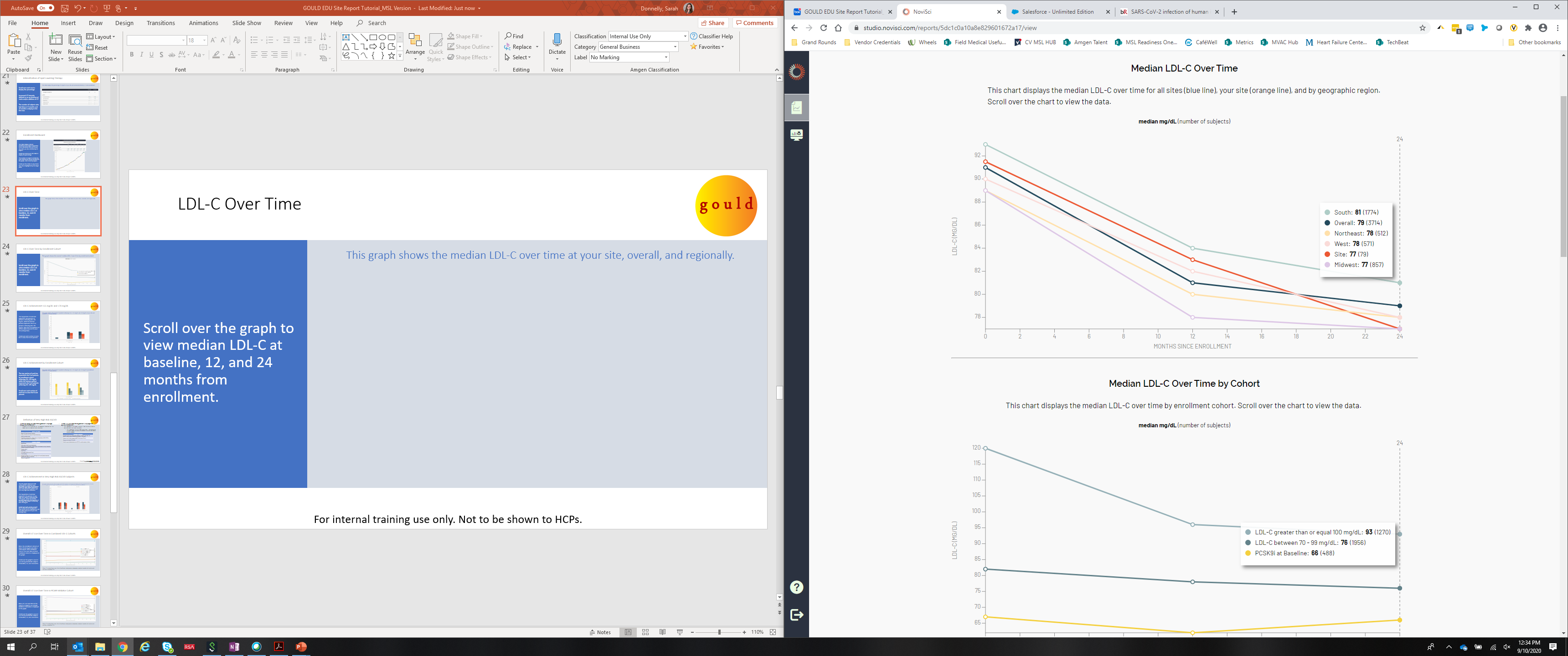
***

***
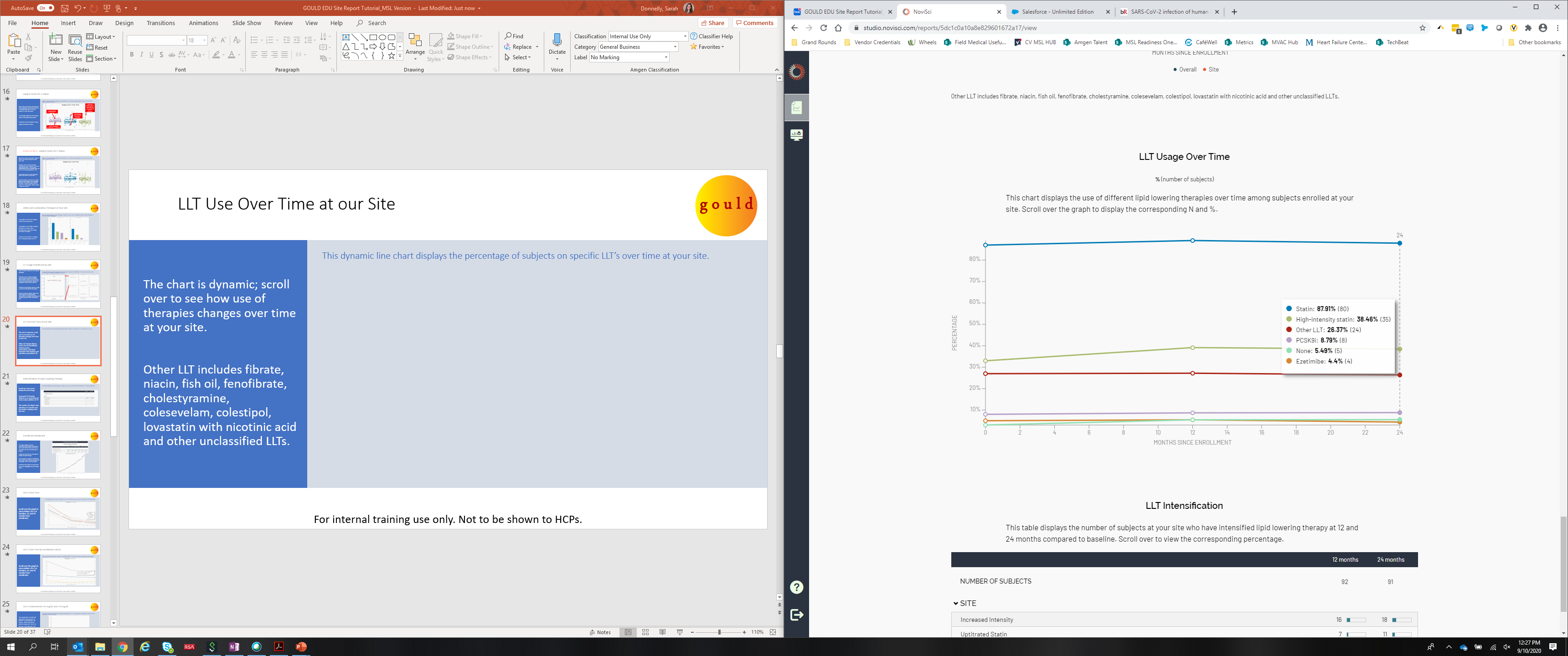
***

Abbreviations: LDL-C, low-density lipoprotein cholesterol; PCSK9i, proprotein convertase subtilisin/kexin type 9 inhibitor.

**Supplemental Figure 3. Patient Flow Diagram**


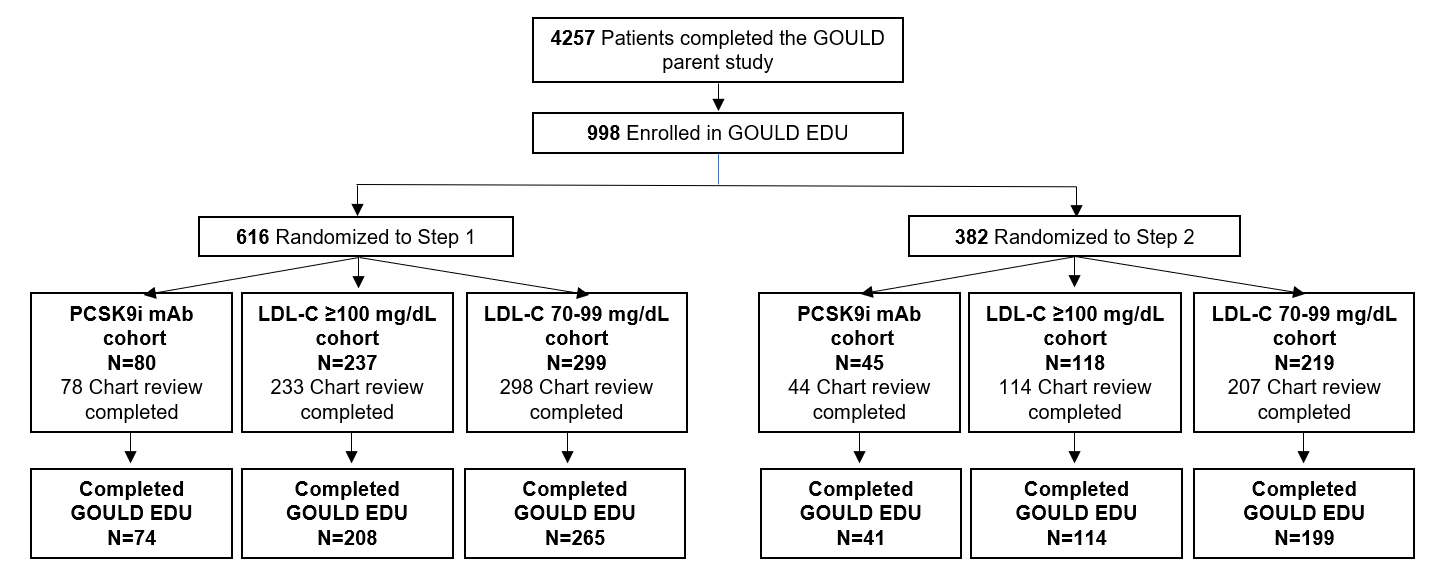


Abbreviations: LDL-C, low-density lipoprotein cholesterol; mAb, monoclonal antibody; PCSK9i, proprotein convertase subtilisin/kexin type 9 inhibitor.

**Supplemental Figure 4. Percentages of Patients With Changes or No Changes in Lipid-Lowering Therapy Intensity During GOULD EDU for Sites Randomized To Step 1 (a) or Step 2 (b)**

**
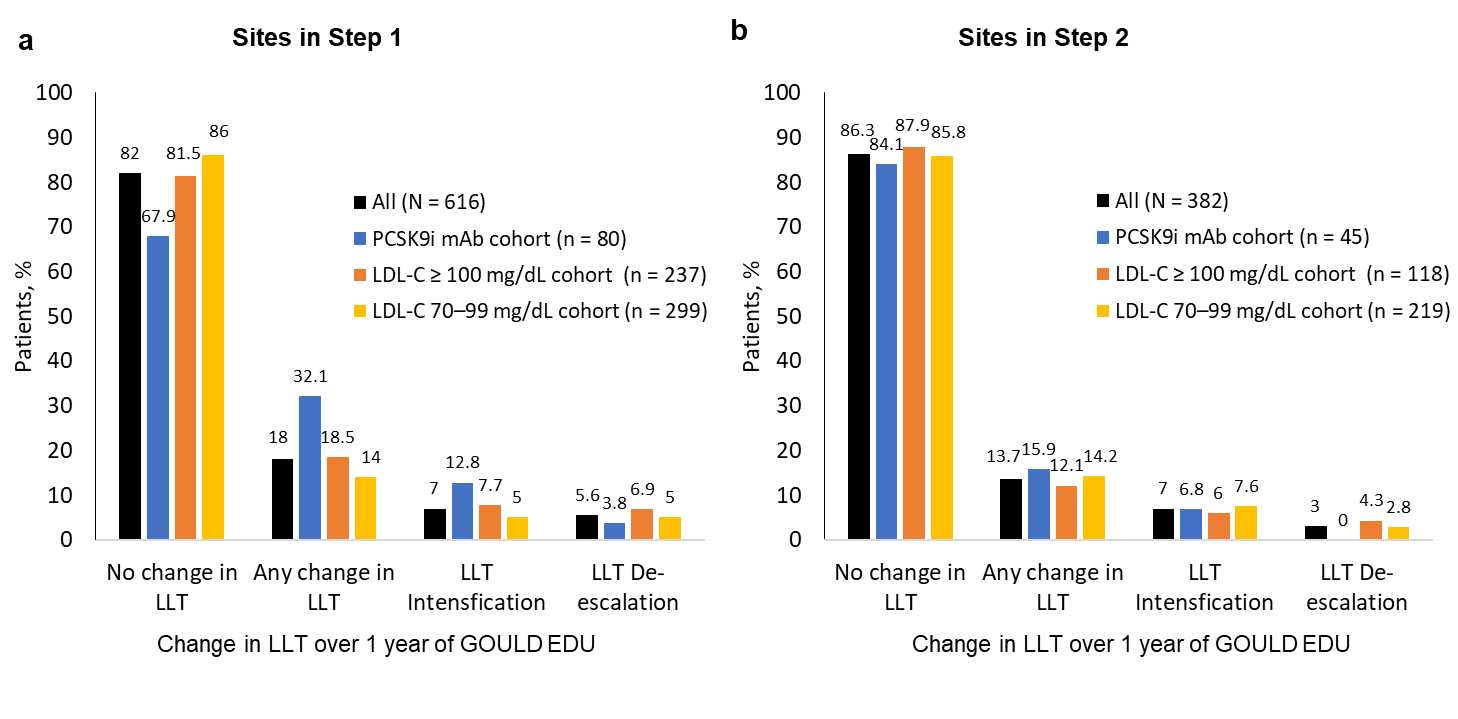
**

Abbreviations: LDL-C, low-density lipoprotein cholesterol; LLT, lipid-lowering therapy; mAb, monoclonal antibody; PCSK9i, proprotein convertase subtilisin/kexin type 9 inhibitor.

**Supplemental Figure 5 A: LDL-C Change Patterns during the GOULD Period and EDU Extension Study Period – Step 1 - LOCF**

| **'** | **PCSK-9i (N=80 Patients)** | **LDL-C>=100 mg/dL (N=237 Patients)** | **LDL-C 70-99 mg/dL (N=299 Patients)** | **Total (N=616 Patients)** |
| --- | --- | --- | --- | --- |
| Baseline |  |  |  |  |
| Mean ± SD (N) | 86.0±53.7 (80) | 130.2±30.5 (237) | 82.1±8.6 (297) | 101.2±36.0 (614) |
| Median (Q1,Q3) | 69.5 (47.0,116.5) | 121.0 (109.0,145.0) | 81.0 (75.0,89.0) | 93.0 (77.0,118.0) |
| Range (Min,Max) | (8.0,273.0) | (46.0,304.0) | (52.0,99.0) | (8.0,304.0) |
| 95% CI | [74.0,97.9] | [126.2,134.1] | [81.1,83.1] | [98.3,104.0] |
| Year 1 |  |  |  |  |
| Mean ± SD (N) | 72.6±51.5 (75) | 97.7±39.1 (170) | 76.5±20.2 (220) | 83.6±35.9 (465) |
| Median (Q1,Q3) | 57.0 (41.0,84.0) | 88.5 (71.0,122.0) | 75.0 (64.0,86.0) | 77.0 (62.0,96.0) |
| Range (Min,Max) | (16.0,321.0) | (21.0,264.0) | (25.0,183.0) | (16.0,321.0) |
| 95% CI | [60.7,84.4] | [91.8,103.7] | [73.8,79.2] | [80.4,86.9] |
| Year 2 |  |  |  |  |
| Mean ± SD (N) | 74.9±37.5 (80) | 98.9±42.6 (202) | 76.6±25.7 (266) | 84.6±36.2 (548) |
| Median (Q1,Q3) | 66.0 (50.0,98.5) | 91.5 (72.0,118.0) | 75.0 (61.0,89.0) | 78.5 (61.0,99.0) |
| Range (Min,Max) | (16.0,170.0) | (10.0,273.0) | (9.0,192.0) | (9.0,273.0) |
| 95% CI | [66.6,83.2] | [93.0,104.8] | [73.5,79.7] | [81.6,87.6] |
| As of June 30, 2020 |  |  |  |  |
| Mean ± SD (N) | 75.7±38.8 (80) | 98.7±42.4 (206) | 77.2±26.7 (279) | 84.8±36.4 (565) |
| Median (Q1,Q3) | 66.0 (46.5,104.5) | 90.5 (71.0,120.0) | 75.0 (60.0,89.0) | 79.0 (61.0,100.0) |
| Range (Min,Max) | (10.0,170.0) | (10.0,277.0) | (9.0,192.0) | (9.0,277.0) |
| 95% CI | [67.1,84.4] | [92.9,104.5] | [74.1,80.4] | [81.8,87.9] |
| At the End of Study |  |  |  |  |
| Mean ± SD (N) | 74.5±42.7 (78) | 98.5±46.3 (212) | 76.9±29.5 (290) | 84.5±39.6 (580) |
| Median (Q1,Q3) | 65.0 (41.0,99.0) | 89.0 (68.5,121.0) | 75.0 (59.0,88.0) | 77.5 (60.5,101.5) |
| Range (Min,Max) | (2.0,200.0) | (7.0,360.0) | (12.0,207.0) | (2.0,360.0) |
| 95% CI | [64.8,84.1] | [92.3,104.8] | [73.5,80.3] | [81.3,87.7] |

| dictall |
| --- |
| LOCF: Last observation carried forward. |
|  |

| PCSK9i Cohort, N= | 80 | 75 | 80 | 80 | 78 |
| --- | --- | --- | --- | --- | --- |
| 70-99 mg/dL Cohort, N= | 297 | 220 | 266 | 279 | 290 |
| >=100 mg/dL Cohort, N= | 237 | 170 | 202 | 206 | 212 |

**Figure 5B: LDL-C Change Patterns during the GOULD Period and EDU Extention Study Period – Step 2 - LOCF**

| **'** | **PCSK-9i (N=45 Patients)** | **LDL-C>=100 mg/dL (N=118 Patients)** | **LDL-C 70-99 mg/dL (N=219 Patients)** | **Total (N=382 Patients)** |
| --- | --- | --- | --- | --- |
| Baseline |  |  |  |  |
| Mean ± SD (N) | 74.8±41.8 (45) | 127.4±30.1 (117) | 81.9±9.1 (218) | 95.1±31.6 (380) |
| Median (Q1,Q3) | 75.0 (38.6,102.0) | 118.0 (107.0,138.0) | 81.0 (75.0,88.0) | 87.5 (76.0,107.0) |
| Range (Min,Max) | (9.0,195.0) | (87.0,275.0) | (65.0,137.0) | (9.0,275.0) |
| 95% CI | [62.3,87.4] | [121.8,132.9] | [80.7,83.2] | [91.9,98.3] |
| Year 1 |  |  |  |  |
| Mean ± SD (N) | 63.4±38.5 (37) | 96.1±33.0 (79) | 79.2±19.4 (153) | 82.0±29.0 (269) |
| Median (Q1,Q3) | 53.0 (38.0,89.0) | 94.0 (74.0,111.0) | 79.0 (66.0,90.0) | 80.0 (63.0,95.0) |
| Range (Min,Max) | (4.0,179.0) | (42.0,239.0) | (25.0,144.0) | (4.0,239.0) |
| 95% CI | [50.6,76.2] | [88.8,103.5] | [76.1,82.3] | [78.5,85.5] |
| Year 2 |  |  |  |  |
| Mean ± SD (N) | 66.4±36.8 (39) | 91.9±35.3 (98) | 76.6±24.3 (189) | 80.0±30.7 (326) |
| Median (Q1,Q3) | 62.0 (36.0,85.0) | 86.0 (66.0,109.0) | 75.0 (64.0,86.0) | 78.0 (62.0,93.0) |
| Range (Min,Max) | (7.0,177.0) | (23.0,208.0) | (6.0,178.0) | (6.0,208.0) |
| 95% CI | [54.4,78.3] | [84.8,99.0] | [73.1,80.1] | [76.6,83.3] |
| As of June 30, 2020 |  |  |  |  |
| Mean ± SD (N) | 64.0±38.1 (44) | 89.8±34.5 (100) | 75.6±22.9 (193) | 78.3±30.1 (337) |
| Median (Q1,Q3) | 56.5 (36.0,87.5) | 84.0 (64.5,106.5) | 75.0 (64.0,86.0) | 76.0 (61.0,92.0) |
| Range (Min,Max) | (7.0,173.0) | (23.0,208.0) | (6.0,161.0) | (6.0,208.0) |
| 95% CI | [52.4,75.6] | [83.0,96.7] | [72.4,78.9] | [75.1,81.6] |
| At the End of Study |  |  |  |  |
| Mean ± SD (N) | 66.8±44.5 (44) | 83.0±33.5 (106) | 73.9±26.3 (194) | 75.8±31.8 (344) |
| Median (Q1,Q3) | 58.5 (32.5,87.0) | 79.5 (60.0,101.0) | 72.0 (60.0,87.0) | 72.0 (56.0,90.0) |
| Range (Min,Max) | (7.0,182.0) | (27.0,183.0) | (6.0,184.0) | (6.0,184.0) |
| 95% CI | [53.3,80.4] | [76.5,89.4] | [70.2,77.7] | [72.5,79.2] |

| dictall |
| --- |
| LOCF: Last observation carried forward. |
|  |

| PCSK9i Cohort, N= | 45 | 37 | 39 | 44 | 44 |
| --- | --- | --- | --- | --- | --- |
| 70-99 mg/dL Cohort, N= | 218 | 153 | 189 | 193 | 194 |
| >=100 mg/dL Cohort, N= | 117 | 79 | 98 | 100 | 106 |

**Figure 5C: LDL-C Change Patterns during the GOULD Period and EDU Extension Study Period – GOULD EDU - LOCF**

| **'** | **PCSK-9i (N=125 Patients)** | **LDL-C>=100 mg/dL (N=355 Patients)** | **LDL-C 70-99 mg/dL (N=518 Patients)** | **Total (N=998 Patients)** |
| --- | --- | --- | --- | --- |
| Baseline |  |  |  |  |
| Mean ± SD (N) | 82.0±49.9 (125) | 129.2±30.4 (354) | 82.0±8.8 (515) | 98.8±34.5 (994) |
| Median (Q1,Q3) | 70.0 (44.0,106.0) | 120.0 (108.0,143.0) | 81.0 (75.0,89.0) | 90.0 (77.0,113.6) |
| Range (Min,Max) | (8.0,273.0) | (46.0,304.0) | (52.0,137.0) | (8.0,304.0) |
| 95% CI | [73.1,90.8] | [126.1,132.4] | [81.3,82.8] | [96.7,101.0] |
| Year 1 |  |  |  |  |
| Mean ± SD (N) | 69.6±47.6 (112) | 97.2±37.2 (249) | 77.6±19.9 (373) | 83.0±33.5 (734) |
| Median (Q1,Q3) | 55.0 (39.5,84.0) | 91.0 (73.0,117.0) | 76.0 (65.0,88.0) | 78.0 (62.0,96.0) |
| Range (Min,Max) | (4.0,321.0) | (21.0,264.0) | (25.0,183.0) | (4.0,321.0) |
| 95% CI | [60.6,78.5] | [92.6,101.9] | [75.6,79.6] | [80.6,85.5] |
| Year 2 |  |  |  |  |
| Mean ± SD (N) | 72.1±37.3 (119) | 96.6±40.5 (300) | 76.6±25.1 (455) | 82.9±34.3 (874) |
| Median (Q1,Q3) | 64.0 (44.0,93.0) | 91.0 (71.0,117.5) | 75.0 (62.0,88.0) | 78.0 (62.0,96.0) |
| Range (Min,Max) | (7.0,177.0) | (10.0,273.0) | (6.0,192.0) | (6.0,273.0) |
| 95% CI | [65.3,78.9] | [92.0,101.2] | [74.3,78.9] | [80.6,85.2] |
| As of June 30, 2020 |  |  |  |  |
| Mean ± SD (N) | 71.6±38.8 (124) | 95.8±40.2 (306) | 76.6±25.2 (472) | 82.4±34.3 (902) |
| Median (Q1,Q3) | 63.0 (41.5,94.5) | 90.0 (70.0,117.0) | 75.0 (61.0,88.0) | 78.0 (61.0,96.0) |
| Range (Min,Max) | (7.0,173.0) | (10.0,277.0) | (6.0,192.0) | (6.0,277.0) |
| 95% CI | [64.7,78.5] | [91.3,100.3] | [74.3,78.9] | [80.2,84.7] |
| At the End of Study |  |  |  |  |
| Mean ± SD (N) | 71.7±43.4 (122) | 93.4±43.0 (318) | 75.7±28.3 (484) | 81.3±37.1 (924) |
| Median (Q1,Q3) | 64.0 (38.0,91.0) | 85.0 (64.0,117.0) | 73.0 (59.0,87.4) | 75.0 (59.0,96.5) |
| Range (Min,Max) | (2.0,200.0) | (7.0,360.0) | (6.0,207.0) | (2.0,360.0) |
| 95% CI | [63.9,79.5] | [88.6,98.1] | [73.2,78.3] | [78.9,83.7] |

| ctall |
| --- |
| LOCF: Last observation carried forward. |


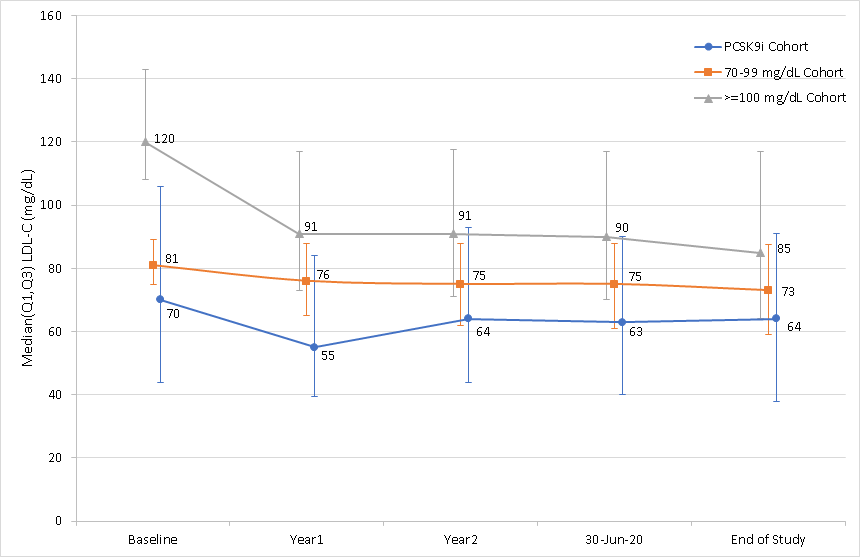


| PCSK9i Cohort, N= | 125 | 112 | 119 | 124 | 122 |
| --- | --- | --- | --- | --- | --- |
| 70-99 mg/dL Cohort, N= | 515 | 373 | 455 | 472 | 484 |
| >=100 mg/dL Cohort, N= | 354 | 249 | 300 | 306 | 318 |
